# Potatoes and risk of chronic disease: a systematic review and dose–response meta-analysis

**DOI:** 10.1007/s00394-018-1774-2

**Published:** 2018-07-09

**Authors:** Lukas Schwingshackl, Carolina Schwedhelm, Georg Hoffmann, Heiner Boeing

**Affiliations:** 1 Department of Epidemiology, German Institute of Human Nutrition Potsdam-Rehbruecke (DIfE), Arthur-Scheunert-Allee 114-116, 14558 Nuthetal, Germany; 2 NutriAct-Competence Cluster Nutrition Research Berlin-Potsdam, Berlin, Germany; 3 Department of Nutritional Sciences, University of Vienna, Althanstrasse 14, UZA II, 1090 Vienna, Austria

**Keywords:** Potatoes, Dose–response, Meta-analysis, Chronic disease

## Abstract

**Purpose:**

We aimed to synthesize the evidence on the relation between different types of potato consumption with risk of all-cause mortality, coronary heart disease (CHD), stroke, type 2 diabetes (T2D), colorectal cancer (CRC), and hypertension.

**Methods:**

Systematic searches until May 2018 were conducted in PubMed, Scopus, and Web of Science. Random effects meta-analyses comparing extreme categories, linear and non-linear dose–response analyses were conducted.

**Results:**

Twenty-eight reports were identified. Only total potato consumption was available for some endpoints which showed no associations with all-cause mortality (RR: 0.88, 95% CI 0.69–1.12), CHD (RR: 1.03, 95% CI 0.96–1.09), stroke (RR: 0.98, 95% CI 0.93–1.03), and CRC (RR: 1.05, 95% CI 0.92–1.20) per one daily/serving (150 g/day) increase. Consumption of one daily serving of boiled/baked/mashed-potatoes was not associated with risk of hypertension (RR: 1.08, 95% CI 0.96–1.21), but slightly with the risk of T2D (RR: 1.09, 95% 1.01–1.18). Positive associations for the risk of T2D (RR: 1.66, 95% CI 1.43–1.94) and hypertension (RR: 1.37, 95% CI 1.15–1.63) were observed for each 150 g/day increase in French-fries consumption. The quality of evidence was rated mostly low (moderate quality of evidence for the risk-associations of French-fries).

**Conclusion:**

Total potato consumption is not related to risk for many chronic diseases but could pose a small increase in risk for T2D if consumed boiled. A clear risk relation was found between French-fries consumption and risk of T2D and hypertension. For several outcomes, the impact of different preparation procedures could not be assessed.

**Electronic supplementary material:**

The online version of this article (10.1007/s00394-018-1774-2) contains supplementary material, which is available to authorized users.

## Background

The present systematic review and meta-analysis was intended to complement previous studies investigating the effects of 12 food groups with respect to risk of all-cause mortality, type 2 diabetes mellitus (T2D), coronary heart disease (CHD), stroke, heart failure, hypertension, and colorectal cancer (CRC). We could show that higher intakes of whole grains, vegetables, fruit, nuts, legumes, dairy, and fish were associated with lower risk of T2D, CHD, stroke, hypertension, and CRC, whereas higher intakes of red and processed meat and SSB were associated with higher risk [1–6].When investigating the interrelationships between diet and health to formulate public health recommendations, foods or food groups should be used in favour of single nutrients. Food groups represent the fundament for food-based dietary guidelines allowing for the consideration of country-specific topics, e.g. dietary preferences or traditions.

Potatoes hold a prominent position among the staples in the Western world. According to data of the Food and Agricultural Organization, 377 Mio. tons of potatoes have been produced worldwide in 2016, approximately 80% thereof in Asia (~ 191 Mio. tons) and Europe (118 Mio. tons) [7].

In prospective observational studies, potato consumption was positively associated with the risk of T2D, hypertension or CRC [8–10]. This is generally attributed to the high content of starch, consecutively leading to a high glycaemic index (GI) [11]. Other components responsible for the detrimental effects of potatoes might be alkaloids or substances synthesized depending on preparation techniques such as acrylamide [12, 13]. On the other side, other prospective observational studies have reported either a neutral association [14, 15] or an inverse association, providing evidence for beneficial effects of regular potato consumption for the prevention of obesity, CVD, or cancer [16, 17]. Potatoes are a considerable source of vitamin C, potassium, fibre, and plant polyphenols [18]. Beneficial or detrimental effects of potatoes might depend to a large extent on various influencing factors such as varieties, cultivation, harvest, or storage. Great variations in preparation and recipes could have a major impact on these constituents as well [18].

A recent systematic review concluded that the included studies do not provide strong evidence of an association between intake of potatoes and risks of obesity, T2D, and CVD, while French fries may be associated with increased risks of obesity and T2D although confounding may have been present [15]. To the best of our knowledge, no meta-analysis has been carried out to date to summarize the association between potato consumption and risk of chronic disease. To be consistent with the studies already carried out [1–6], we aimed to meta-analyse the association between consumption of different types of potato with the risk of either all-cause mortality, CHD, stroke, hypertension, CRC, or T2D. To explore the shape of the relationship, we explored highest vs. lowest intake categories as well as linear and non-linear relationships. In addition, the quality of evidence was investigated using the NutriGrade scoring system.

## Methods

The following meta-analysis is part of a systematic review protocol and was registered in PROSPERO (http://www.crd.york.ac.uk/prospero/index.asp, identifier CRD42016037069). Our strategy for the present systematic review is an extension of a pre-defined and published protocol, [19] and has already been implemented by several published meta-analyses investigating the association between 12 food groups and risk of all-cause mortality [4], T2D [2], CHD, stroke, heart failure [1], hypertension [3], and CRC [5]. This meta-analysis followed the guidelines for reporting proposed by the Meta-analyses Of Observational Studies in Epidemiology (MOOSE) [20].

### Search strategy

PubMed, Web of Science, and Scopus were searched until May 2018. The full search strategy for PubMed is given in ESM Material 1.

Manual searches included the reference lists from the identified studies. Two authors (LS, CS) performed the literature search, while another author (GH) reviewed uncertain cases. Consensus was reached through discussion between both authors.

### Study selection

As previously described, we included studies with cohort, case–cohort, and nested case–control design, as well as follow-ups of RCTs. Included studies investigated the association between potato intake (total potato, French fries, and boiled/baked/mashed potato consumption) on risk of all-cause mortality, CHD, stroke, heart failure, CRC, T2D, and hypertension in adults (≥ 18 years). The definitions of the corresponding chronic diseases were based on the previously published meta-analyses [1–5].

### Data extraction

The following data were extracted for each study: name of first author, publication year, country, cohort name, sample size, number of cases, baseline age, sex, duration of follow-up, specification of outcome assessment, specification of type of potato consumed, method of dietary assessment, quantity of potato intake, multivariable effect estimate with corresponding 95% confidence intervals (CIs), and covariates. When the risk estimates for participants were reported only separately for men and women in a study, the risk ratios (RRs) were combined using a fixed effect model.

### Statistical analysis

We applied a random effects model [21] to derive summary RRs and 95% CIs, investigating the associations between the categories of highest vs. lowest intake and the dose–response estimate for potato consumption (total potato, French fries, and boiled/baked/mashed potato consumption) and risk of all-cause mortality, CHD, stroke, heart failure, CRC, T2D and hypertension. We calculated the standard error for the logarithm RR of each study using an inverse variance method. This was in turn considered the estimated variance of the logarithm RR [21]. The meta-analysis was based on the assumption that all measures are RRs. We applied the method described by Greenland and Longnecker for the dose–response analysis [22, 23]. The distribution of cases and person-years or non-cases, as well as the RRs with the 95% CI were required for at least three quantitative exposure categories for the application of this method. If directly reported in an identified study, a linear dose–response estimated with 95% CI was directly included in our analyses. Meta-analyses were conducted if ≥ 3 studies were available for each corresponding outcome.

As described previously, if studies reported only total number of cases or person-years and the exposure was defined in categories, the number of person-years or cases in each category was obtained from the total number of person-years/cases divided by the number of reported categories. We assigned the median or mean intake by quantile to the corresponding risk estimate. If studies reported intakes only as a range by quantile, the midpoint was calculated. In the case of an open-ended intake range, we assumed that the width was the same as the contiguous category. If the exposure was expressed per given unit of energy intake, we used the provided mean energy intake to rescale it.

The dose–response was expressed as 150 g/day (weight of a medium potato). If studies reported exposure only in serving size but not specified the amount, 150 g/day as serving was used.

Restricted cubic splines for each study with more than three quantiles of exposure were calculated to explore possible nonlinear associations. We used three fixed knots through the total range of the reported intake at 10, 50, and 90% and combined these using multivariate meta-analysis [24].

Heterogeneity between studies was evaluated using the *Q* test and the *I*^2^ statistic. A value greater than 50% for the *I*^2^ statistic was regarded as potentially important statistical heterogeneity [25]. If more than five studies were available for an outcome in the linear dose–response analysis, subgroup analyses were performed by the following characteristics: sex, length of follow-up (mean or median ≥ 10 vs. <10 years), geographic location (by continent), number of cases (≥ 1000 vs. <1000), and validated/non-validated dietary assessment to identify potential sources of heterogeneity.

As recommended by the Cochrane Handbook, if 10 or more studies were available [26], we explored potential small-study effects such as publication bias using Egger’s test and funnel plots [27]. Stata version/SE 14.2 software (StataCorp, College Station, TX, USA) and Review Manager 5.3 (Nordic Cochrane Centre, Copenhagen) were used to conduct statistical analyses.

### Quality of meta-evidence

To evaluate the quality of evidence for the association between potato consumption and risk of all-cause mortality, CHD, stroke, heart failure, T2D, CRC, and hypertension we applied our recently developed NutriGrade scoring system (max 10 points) [28]. This tool is based on the following eight items for cohort studies: (1) risk of bias, study quality, study limitations (max. 2 points), (2) precision (max. 1 point), (3) heterogeneity (max. 1 point), (4) directness (max. 1 point), (5) publication bias (max. 1 point), (6) funding bias (max. 1 point), (7) effect size (max. 2 points), and (8) dose–response (max. 1 point) [28]. To evaluate and interpret the meta-evidence, we recommend four categories based on this scoring system: high (≥ 8 points), moderate (6–< 8 points), low (4–< 6 points), and very low (0–< 4 points).

## Results

Out of the 556 records which were identified by the literature search, 95 full text articles were assessed in detail (ESM Fig. 1, ESM Material 2).

Six prospective cohort studies were included for all-cause mortality [29–34], seven prospective cohort studies (five reports) for CHD [14, 35–38], six prospective cohort studies (four reports) for stroke [14, 37, 39, 40], two prospective cohort studies (one report) for heart failure [14], eight prospective cohort studies (seven reports) for CRC [41–47], eight prospective studies (seven prospective cohort studies, one RCT analysed as prospective cohort study) (six reports) for T2D [48–53], and four studies (two reports) for hypertension [10, 54] (ESM Table 1).

### All-cause mortality

Six studies with 26,775 death cases were included in the highest vs. lowest intake category meta-analysis (overall intake range 0–184 g/day). Five of these studies reported total potato consumption [29–31, 33, 34], and one study reported only fried potato consumption [32]. No association between all-cause mortality and total potato intake was observed (RR: 0.97; 95% CI 0.86, 1.10, *I*^2^ = 61%, *p*_heterogeneity_ = 0.03) when comparing extreme categories (ESM Fig. 2). Similarly, an increase in overall potato intake by 150 g per day was not associated with risk of all-cause mortality (RR: 0.88; 95% CI 0.69, 1.12, *I*^2^ = 81%, *p*_heterogeneity_ < 0.001, *n* = 5) (ESM Fig. 3).

**Fig. 1:**
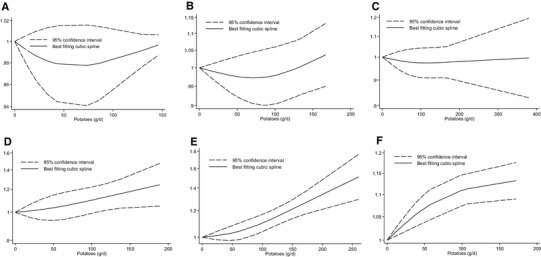
Non-linear dose–response relation between daily intakes of total potato consumption and risk of all-cause mortality (**a**) (*p*_non-linearity_ = 0.25; *n* = 4 studies), coronary heart disease (**b**) (*p*_non-linearity_ = 0.18; *n* = 5 studies), stroke (**c**) (*p*_non-linearity_ = 0.53; *n* = 6 studies), colorectal cancer (**d**) (*p*_non-linearity_ = 0.69; *n* = 6 studies), type 2 diabetes (**e**) (*p*_non-linearity_ = 0.14; *n* = 6 studies) and hypertension (*p*_non-linearity_ = 0.06; *n* = 4 studies) (**f**)

There was no evidence of a non-linear dose–response association (*p*_non-linearity_ = 0.25, *n* = 4 studies) (Fig. 1).

### Coronary heart disease and stroke

Six studies with 8586 coronary heart disease cases, and six studies with 6902 stroke cases were included in the highest vs. lowest intake category meta-analysis (range of intake 0–212 g/day). All of these studies reported total potato consumption [14, 35–37, 39, 40] but one article reported information on the type of potato consumption (no association was observed between boiled, and fried potatoes or French fries and the risk of CHD, HF or stroke) [14]. Comparing categories of highest vs. lowest intake of total potato consumption, we observed no association with risk of CHD (RR: 1.02; 95% CI 0.95, 1.09, *I*^2^ = 0%, *p*_heterogeneity_ = 0.96) and stroke (RR: 0.98; 95% CI 0.88, 1.08, *I*^2^ = 27%, *p*_heterogeneity_ = 0.23) (ESM Fig. 4). Similarly, an increase in total potato intake by 150 g per day was not associated with risk of CHD (RR: 1.03; 95% CI 0.96, 1.09, *I*^2^ = 0%, *p*_heterogeneity_ = 0.99, *n* = 7) or stroke (RR: 0.98; 95% CI 0.93, 1.03, *I*^2^ = 3%, *p*_heterogeneity_ = 0.40, *n* = 6) (ESM Fig. 5).

In additional analyses stratified by sex, follow-up duration, geographic location, number of cases, and dietary assessment method no statistically significant subgroup differences were observed (ESM Tables 2, 3).

No evidence of a non-linear dose–response association was detected for CHD (*p*_non-linearity_*p* = 0.18, *n* = 5 studies) and stroke (*p*_non-linearity_*p* = 0.53, *n* = 6 studies) (Fig. 1).

### Colorectal cancer

Eight studies including 3567 incident colorectal cancer cases were included in the meta-analysis comparing extreme intake categories (range of intake 0–221 g/day). Six studies reported total potato consumption [41–45], one study reported only pan-fried potato consumption [46], and one study reported only sweet potato intake [47]. We observed no association between risk of CRC and total potato intake in the high vs. low meta-analysis (RR: 1.13; 95% CI 0.96, 1.33, *I*^2^ = 52%, *p*_heterogeneity_ = 0.05) (ESM Fig. 6) or in the dose–response meta-analysis (RR per 150 g/day: 1.05; 95% CI 0.92, 1.20, *I*^2^ = 20%, *p*_heterogeneity_ = 0.28, *n* = 6) (ESM Fig. 7).

In additional analyses statistically significant subgroup differences for total potato consumption were observed for length of follow-up, and geographic location (ESM Table 4). Stronger positive associations were observed for studies conducted in the Europe, and in studies with long-term follow-up (≥ 10 years).

There was no evidence of a non-linear dose–response trend (*p*_non-linearity_ = 0.69, *n* = 6 studies). For a daily total potato intake greater than 134 g, the risk of CRC increased by approximately 25% up to ~ 190 g/day (Fig. 1).

### Type 2 diabetes mellitus

Seven studies with 18,334 incident T2D cases were included in the meta-analysis comparing extreme intake categories (range of intake 0–326 g/day). Four of these seven studies provided additional information for French fries and boiled/baked/mashed potato consumption [51, 52]. We observed a positive association between risk of T2D and total potato consumption (RR: 1.19; 95% CI 1.07, 1.32, *I*^2^ = 36%, *p*_heterogeneity_ = 0.15) (ESM Fig. 8). An increase in total potato intake by 150 g per day was positively associated with risk of T2D (RR: 1.18; 95% CI 1.10, 1.27, *I*^2^ = 30%, *p*_heterogeneity_ = 0.20, *n* = 7) (ESM Fig. 9). However, considering only French fries consumption (per 150 g/day) showed a considerable stronger positive association with T2D risk (RR: 1.66, 95% CI 1.43, 1.94; *I*^2^ = 0%), whereas boiled/baked/mashed potato consumption showed only a slightly increased risk of T2D (RR: 1.09, 95% CI 1.01, 1.18; *I*^2^ = 0%) (ESM Fig. 10) (Test for subgroup difference; *p* < 0.001).

In additional analyses statistically significant subgroup differences for total potato consumption were observed for length of follow-up, geographic location, and dietary assessment (ESM Table 5). Stronger positive associations were observed for studies conducted in the US, in studies with long-term follow-up (≥ 10 years), and in studies applying validated dietary assessment methods.

There was no evidence of a non-linear dose–response trend (*p*_non-linearity_ = 0.14, *n* = 6 studies). The risk of T2D increased by approximately 51% (predominately due to French fries consumption) with increasing intake of potatoes up to ~ 260 g/day (Fig. 1).

### Hypertension

Four studies with 78,484 incident hypertension cases were included in the meta-analysis comparing extreme intake categories (range of intake 0–172 g/day). All of these studies provided additional information about the types of potato consumed (French fries vs. baked/oiled/mashed potatoes). No association between risk of hypertension and total potato intake was observed (RR: 1.09; 95% CI 0.92, 1.29, *I*^2^ = 71%, *p*_heterogeneity_ = 0.02) when comparing extreme categories (ESM Fig. 11). An increase in total potato intake by 150 g per day was positively associated with risk of hypertension (RR: 1.12; 95% CI 1.01, 1.23, *I*^2^ = 87%, *p*_heterogeneity_ < 0.001, *n* = 4) (ESM Fig. 12). However, only French fries consumption (per 150 g/day) showed a positive association with risk of hypertension (RR: 1.37, 95% CI 1.15, 1.63; *I*^2^ = 69%), whereas boiled/baked/mashed potato consumption showed no association (RR: 1.08, 95% CI 0.96, 1.21; *I*^2^ = 84%) (ESM Fig. 13) (test for subgroup difference; *p* = 0.03).

Some evidence of a non-linear dose–response association was observed (*p*_non-linearity_ = 0.06, *n* = 4 studies). The risk of hypertension increased by approximately 13% (predominately due French fries consumption) with increasing intake of potatoes up to ~ 170 g/day (Fig. 1).

### Quality of evidence

Overall, the credibility of evidence for the association between total potato intake and risk of all-cause mortality, CHD, stroke, CRC, T2D and hypertension was rated “low”, whereas the quality of evidence for the association between French fries consumption and risk of T2D and hypertension was rated as “moderate” (ESM Table 6).

## Discussion

In the present meta-analysis, we observed a positive association between French fries consumption and risk of T2D as well as hypertension following linear dose–response relations, whereas boiled/baked/mashed potato consumption seemed to have negligible influence on these outcomes. For all other outcomes, we were only able to investigate total potato consumption, which resulted in a neutral association. Findings were rated as low quality of evidence (all-cause mortality, CHD, stroke, CRC, hypertension, T2D) for total potato consumption, and moderate quality of evidence for French fries consumption (T2D and hypertension).

According to the literature, the GI represents one mechanism to explain the detrimental associations of French fries consumption [55]. Although the GI of French fries is usually lower than the one in cooked or baked variants, they still contribute to the glycaemic load, which has been associated with increased risk of T2D [56]. However, the relevance of simple GI classification is discussed controversially in the literature. Variations in GI values can already be observed when considering different cultivars [57]. Thus, potatoes contain additional constituents that may affect the utilization of starch in the human organism, e.g. varieties rich in polyphenols have been demonstrated to inhibit the catalytic cleavage of starch in the small intestine [58, 59], and an inverse association between potato phenol content and GI has been found [60]. Moreover, deep frying is a form of preparation that can significantly increase the fat content of the meal which may consecutively promote the onset of overweight, obesity, and T2D [15, 61]. Moreover, the quality of fat is mostly inadequate (e.g. via formation of trans-fat).

Usually, potatoes are part of a complex meal [17], and this could implicate the simultaneous intake of unfavourable food groups, e.g. red or processed meat as an additional source of sodium chloride as well as sugar-sweetened beverages as an additional source of empty calories [8]. This is often accompanied by different preparation procedures (boiled potatoes/healthy pattern − French fries/Western diet) [62]. In the US, fresh potatoes are being replaced more and more by processed variants. This could at least in part explain the results of our geographical subgroup analyses revealing a more important association between potato intake and T2D in the US as compared to other regions [63].

By applying substitution models it was shown that substituting one serving a day of baked/boiled/mashed potato for non-starchy vegetables was associated with a 7% reduced risk of hypertension in three US studies [10]. Moreover, substituting three servings weekly of baked/boiled/mashed potato, or French fries for whole grains was associated with a 5 or 17% reduced risk of T2D [51]. In the Danish Diet, Cancer and Health study substituting 150 g of red meat per week for potatoes was associated with a lower risk of myocardial infarction [38]. Unfortunately, no substitution analyses to exchange potatoes with noodles or rice have been conducted.

The beneficial effects of potatoes on blood pressure due to their high potassium content [64] is thwarted by the addition of sodium chloride to fried products, which might accelerate the development of hypertension. In our systematic review, we could not establish whether deep-fried potatoes were manufactured using sophisticated methods that can minimize these unwanted side effects, e.g. coating technologies, vacuum frying, or application of MUFA-rich oils to reduce trans fats [65, 66].

Regardless of components with potential adverse effects on human health, potatoes belong to the most frequently consumed plant-based food groups worldwide. One of the controversies concerns the question of whether they should be regarded as vegetables or not. While recommendations such as “MyPlate” in the US take this stance, potatoes belong to cereals according to the British “EatwellPlate” guidelines [67]. This also affects health care initiatives aimed at specific population groups. Initially, the US School Lunch Program *Healthy, Hunger-Free Act of 2010* implemented a restriction of vegetables rich in starch to one portion per day, which was ultimately withdrawn in 2012 [68]. Likewise, in the original 2009-version of the Special Supplemental Nutrition Program for Women, Infants, and Children, white potatoes were expressively left out of the (food-based) recommendations, which was cancelled in 2015 [69].

The above-mentioned discrepancies between international recommendations can in part be explained by evidence considering the association of different types of potato intake with health outcomes such as T2D or hypertension [8, 70], as observed in the present meta-analysis. T2D and hypertension present a serious threat for public health. Prevalence of T2D has been estimated to 425 Mio. individuals in 2017, and future numbers have been projected to increase to almost 629 Mio. patients by 2045 [71]. Approximately 40% of individuals older than 25 years are considered to be hypertensive, which equalled to 1 billion people in 2008. As with T2D, the prevalence continues to rise [72].

Figuratively speaking, potatoes might be regarded as a double-edge sword in this context. Besides their potential detrimental effects described above, potatoes are considered to be easily available suppliers of vital substances such as, e.g. fibre, potassium, vitamin C and secondary plant metabolites. With an average fibre content of approximately 2 g/100 g, potatoes are not among the predominant sources of dietary fibre, however, they become relevant when taking into account the usual amount of consumption as well as the impact of specific preparation procedures which might increase RS/fibre content [73]. Due to their high potassium content, potatoes are no longer stigmatized as a simple starch-containing vegetable in the context of the School Lunch Program Healthy *Hunger-Free Act of 2010* but instead considered as a valuable source for the mineral [68, 74]. Despite micronutrients such as vitamin C and selenium, the phenol fraction seems to be the most important contributor to the antioxidant capacity of potatoes [75].

### Strengths and limitations

Several limitations should be acknowledged in the interpretation of the results of the present meta-analysis. Summing up, only a very limited number of prospective observational studies were identified for two out of six outcomes (< 6 studies for all-cause mortality, and hypertension). Moreover, only few studies distinguished between potato preparation methods (i.e. French fries vs. baked/boiled/mashed potatoes) and reported its association with risk of chronic disease. These are clear limitations, since substantial differences exist with regards to energy density of carbohydrate and fat quantity within similar portion sizes across preparation methods [76]. Possible confounding from unhealthy food choices and lifestyle behaviours that were associated with the type of potato preparation should be acknowledged. Caution needs to be taken when interpreting our findings, since also preparation methods vary widely across different populations.

Strengths of this systematic review are the large number of studies enrolled for meta-analyses and the implementation of different types of meta-analyses to explore highest vs. lowest intake categories as well as linear and non-linear dose–response relationships. In addition, we investigated the quality of evidence using the NutriGrade score. However, there are limitations that should be taken into account when interpreting our findings.

## Conclusions

Potato consumption in general is not related to risk for many chronic diseases but could pose a small increase in risk for T2D if consumed boiled. A clear risk relation was found between French fries consumption and risk of T2D and hypertension. One of the main limitations in the interpretation of the data is the fact that the impact of different preparation procedures on ingredients exerting these detrimental effects could not be assessed in the studies enrolled in our meta-analyses. Considering the importance of this food group for human nutrition in many parts of the world, potatoes should not be prematurely condemned as an unhealthy part of our diet. Their evaluation and use as a source of valuable nutrients in dietary recommendations should rather take into consideration the progress in the field of cultivation and preparation techniques.

## Electronic supplementary material

Below is the link to the electronic supplementary material.

Supplementary material 1 (PDF 526 KB)

## Abbreviations

CHD: Coronary heart disease
CRC: Colorectal cancer
CVD: Cardiovascular disease
GI: Glycaemic index
RR: Risk ratio
RCTs: Randomized controlled trials
T2D: Type 2 diabetes
