# Supplementary material for: Potatoes and risk of chronic disease: a systematic review and dose–response meta-analysis
Source: Eur J Nutr. 2018 Jul 9;58(6):2243–51. doi: 10.1007/s00394-018-1774-2 (PMC6689281; doi:10.1007/s00394-018-1774-2)
Supplement: Supplementary file 1 — Supplementary material 1 (PDF 526 KB) [file 394_2018_1774_MOESM1_ESM.pdf]

Potatoes and risk of chronic disease: a systematic review and dose-response meta-analysis

Lukas Schwingshackl[1,2], Carolina Schwedhelm[1,2], Georg Hoffmann[3], Heiner Boeing[1,2]

[1] Department of Epidemiology, German Institute of Human Nutrition Potsdam-Rehbruecke (DIfE), Arthur-Scheunert-Allee 114-116, 14558 Nuthetal, Germany

[2] NutriAct – Competence Cluster Nutrition Research Berlin-Potsdam

[3] Department of Nutritional Sciences, University of Vienna, Althanstraße 14, UZA II, 1090 Vienna, Austria

Corresponding author: Lukas Schwingshackl, PhD

Arthur-Scheunert-Allee 114-116; 14558 Nuthetal, Germany

M: lukas.schwingshackl@dife.de

T: +49 (0)33200 88-2723

Abbreviations:

7TT: Seven Townships Taiwan

95% CI: 95% confidence interval

aHEI: alternate Healthy Eating Index

AHS: Adventist Health Study

BCDDP: Breast Cancer Demonstration Project) follow-up study cohort

BMI: Body Mass Index

C: Colon

CHD: Coronary heart disease

COSM: Cohort of Swedish Men

CRC: Colorectal cancer

DASH: Dietary approaches to Stop Hypertension

DDCHS: Danish Diet, Cancer and Health Study

EPIC: European Prospective Investigation Into Cancer and Nutrition

FMCHES: Finnish Mobile Clinic Health Examination Survey

FFQ: Food frequency questionnaire

HF: Heart failure

HR: Hazard ratio

HPFS: Health Professional Follow-up study

IWHS: Iowa Women's Health Study

M: Men

MCCS: Melbourne Collaborative Cohort Study

MDCS: Malmö Diet and Cancer Study

MI: Myocardial infarction

MICOL: Multicentrica Italiana Colelitiasi, Italian, Multicentric study for cholelithiasis

NHS: Nurses' Health Study

NOWAC: Norwegian Women and Cancer study

NUTRIHEP: Nutrition Hepatology Study

NYUWHS: New York University Women´s Health Study

OICS: Osteoarthritis Initiative Cohort Study

OR: Odds ratio

R: Rectum

RR: Risk ratio

SDASE: Study on Diet, antioxidant status and survival in the elderly

SMC: Sweden Mammography cohort

SUN: Seguimiento University of Navarra

SWHS: Shanghai Women's Health Study

T2D: Type 2 diabetes mellitus

US: United States

W: Women

WHS: Women's Health Study

ESM Material 1:

Search strategy (PubMed):

#1 potato*[tiab] OR solanum tuberosum[tiab] OR french fry[tiab] OR french fries[tiab] OR potato chip*[tiab]

#2 mortality[tiab] OR death[tiab]

#3 cardiovascular[tiab] OR coronary[tiab] OR stroke[tiab] OR vascular[tiab] OR myocardial infarction[tiab] OR heart failure[tiab]

#4 colon[tiab] OR rectum[tiab] OR rectal[tiab] OR colorectal[tiab] OR colorectum[tiab] OR bowel[tiab]

#5 diabetes[tiab]

#6 hypertension[tiab] OR hypertensive[tiab] OR systolic[tiab] OR diastolic[tiab] OR blood pressure[tiab]

#7 prospective OR cohort OR longitudinal OR follow-up OR case-cohort OR nested case-control

#8 (#1 AND #2 OR #3 OR #4 OR #5 OR #6 AND #7)

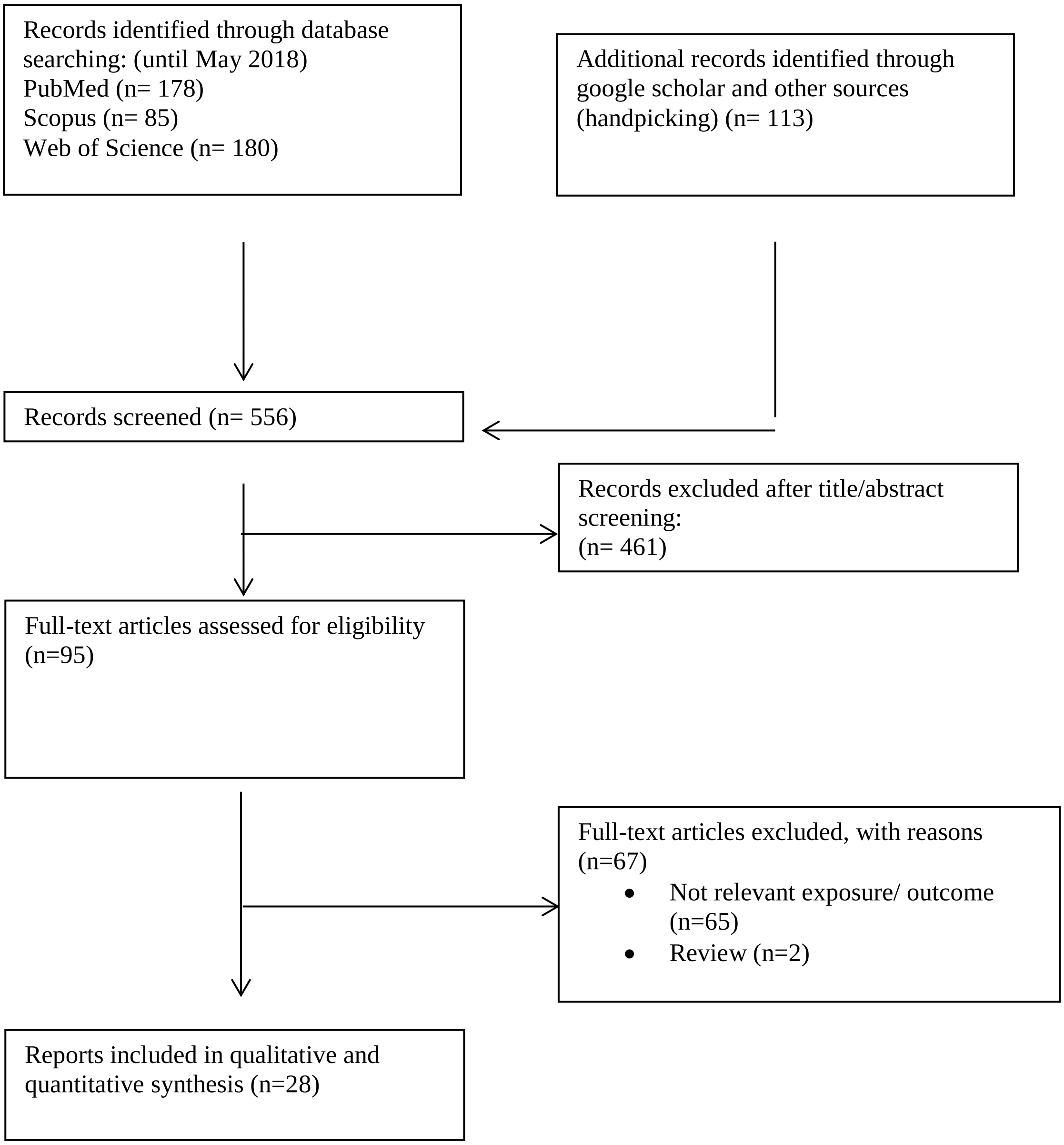

ESM Figure 1: Flow diagram for study selection

ESM Material 2: Full-text publications excluded with reason

(1-65)	Not relevant exposure/ outcome
(66, 67)	Reviews

ESM Table 1: General study characteristics of the included studies investigating the association between potatoes intake and risk all-cause mortality, coronary heart disease, stroke, heart failure, colorectal cancer, type 2 diabetes, and hypertension.

**First Author**	**Year**	**Country**	**Cohort Name**	**Age at entry, y**	**Sex**	**Sample size, n**	**Total cases, n**	**Dietary assessment**	**Outcome**	**Outcome assessment**	**Type potatoes**	**Adjustment factors**	**Follow up years**	**Results (high vs. low intake category) RR/HR/OR (95% CI)**
Asli	2016	Norway	NOWAC	30-70	W	*95,942*	*912*	FFQ, validated	CRC	Linkage to the Cancer Registry of Norway	Total potato consumption (usually boiled potatoes)	Education, smoking, red meat, milk, non-potato fibre, processed meat, hormone replacement therapy, carbohydrate energy, and non-carbohydrate energy, pasta, rice, fruit, and vegetables.	12	1.30 (1.07, 1.57)<br><br>3 vs. 1 tertile
Borgi	2016	US	NHS	30-55	W	*62,175*	*35,375*	FFQ, validated	Hypertension	Self-reported (validated)	Total potato consumption; Baked, boiled, or mashed potatoes; French fries	Age, race/ethnicity, BMI, smoking status, physical activity, weight change, menopausal status, alcohol intake, analgesic use, family history of hypertension, total energy intake, animal flesh intake, whole grains, sugar sweetened drink intake, artificially sweetened diet drink intake, total fruit, total vegetables	1,034,421 person years	Total potato consumption: 1.07 (0.92, 1.24)<br><br>5 vs. 1 quintile<br><br>Baked, boiled, or mashed: 1.13 (1.02, 1.26)<br><br>4 vs. 1 quartile<br><br>French fries: 1.17 (0.92, 1.50)<br><br>4 vs. 1 quartile
Borgi	2016	US	NHS II	25-42	W	*88,475*	*25,246*	FFQ, validated	Hypertension	Self-reported (validated)	Total potato consumption;	Age, race/ethnicity, BMI, smoking	1,344,475 person	Total potato consumption: 1.42

											Baked, boiled, or mashed potatoes; French fries	status, physical activity, weight change, menopausal status, alcohol intake, current oral contraceptive use, analgesic use, family history of hypertension, total energy intake, animal flesh intake, whole grains, sugar sweetened drink intake, artificially sweetened diet drink intake, total fruit, total vegetables	years	(1.18, 1.72)<br><br>5 vs. 1 quintile<br><br>Baked, boiled, or mashed:<br>1.25 (1.11, 1.41)<br><br>4 vs. 1 quartile<br><br>French fries:<br>1.17 (1.04, 1.33)<br><br>4 vs. 1 quartile
Borgi	2016	US	HPFS	40-75	M	*36,803*	*16,752*	FFQ, validated	Hypertension	Self-reported (validated)	Total potato consumption; Baked, boiled, or mashed potatoes; French fries	Age, race/ethnicity, BMI, smoking status, physical activity, weight change, alcohol intake, analgesic use, family history of hypertension, total energy intake, animal flesh intake, whole grains, sugar sweetened drink intake, artificially sweetened diet drink intake, total fruit, total vegetables	560,228 person years	Total potato consumption:<br>0.96 (0.79, 1.16)<br><br>5 vs. 1 quintile<br><br>Baked, boiled, or mashed:<br>0.95 (0.84, 1.08)<br><br>4 vs. 1 quartile<br><br>French fries:<br>1.16 (1.00, 1.33)<br><br>4 vs. 1 quartile
Dilis	2012	Greece	EPIC-Greece	20-86	Both	*23,929*	*636*	FFQ, validated	CHD	Self-reported/confirmed through medical records	Potatoes	Age, BMI, height, physical activity, years of schooling, energy intake, alcohol consumption, smoking, arterial	10	1.01 (0.91, 1.12)<br><br>Per 1 SD increment

												blood pressure, other nutritional variables		
Flood	2002	US	BCDDP	60	W	*45,490*	*314*	FFQ, validated	CRC	Self-reported, phone calls, mailing, medical and pathology records	Potatoes (except French fries)	Multivitamin supplement use, BMI, height, use of nonsteroidal anti-inflammatory drugs, smoking status, education level, physical activity, and intakes of grains, red meat, calcium, vitamin D, and alcohol	8.6	0.82 (0.65, 1.15)<br><br>5 vs. 1 quintile
Gonzalez	2008	Spain	SDASE	60-85	Both	*288*	*83*	FFQ	All-cause mortality	Death registers	Potatoes	Age, sex, BMI, energy intake, hyperglycaemia, hypercholesterolemia, chewing ability, physical activity, smoking, self-perceived health, education level, institution	6	1.32 (1.03, 1.69)<br><br>Per 1 SD increment
Hodge	2004	Australia	MCCS	40-69	Both	*31,641*	*365*	FFQ	T2D	Self-reported/ doctor confirmation	Potatoes	Age, sex, BMI, waist: hip ratio, energy intake, education, alcohol, physical activity, weight change in the last 5 years, country of birth, family history of diabetes	4	0.98 (0.70, 1.37)<br><br>4 vs. 1 quartile
Hu	2017	Spain	SUN	36	Both	*13,837*	*1,111*	FFQ	Hypertension	Self-reported and validated	Total potato consumption; Cooked and roasted potatoes; Fried potatoes	Age, sex, and recruitment period as well as for BMI, family history of hypertension, physical activity, smoking, education,	6.7	Total potato consumption: 0.98 (0.80, 1.19)<br><br>5 vs. 1 quintile

												television watching, total energy intake, sodium intake, consumption of fast food, low-fat dairy products, fruits, vegetables, tree nuts, fried foods, alcohol, olive oil, and sugar-sweetened beverages		Cooked/ roasted potatoes: 0.88 (0.73, 1.07)<br><br>5 vs. 1 quintile<br><br>Fried potatoes: 0.92 (0.75, 1.13)<br><br>5 vs. 1 quintile
Joshipura	1999	USA	NHS	34-59	W	*75,596*	*366*	FFQ, validated	Ischaemic stroke	Self-reported/questionnaire; medical records	Potatoes	Age, smoking, alcohol, family history of myocardial infarction, BMI, vitamin supplement use, vitamin E use, physical activity, aspirin use, 7 time periods for women, hypertension and hypercholesterolemia, total energy intake, postmenopausal hormone use	14	1.09 (0.69, 1.72)<br><br>5 vs. 1 quintile
Joshipura	1999	USA	HPFS	40-75	M	*38,683*	*204*	FFQ, validated	Ischaemic stroke	Self-reported/questionnaire; medical records	Potatoes	Age, smoking, alcohol, family history of myocardial infarction, BMI, vitamin supplement use, vitamin E use, physical activity, aspirin use, 7 time periods for women, hypertension and hypercholesterolemia, total energy intake, postmenopausal	8	1.23 (0.88, 1.72)<br><br>5 vs. 1 quintile

												hormone use		
Joshipura	2001	USA	NHS	34-59	W	*84,251*	*1,127*	FFQ, validated	CHD	Records were reviewed by physicians who were blinded to the participants' risk factor status.	Potatoes	Age, smoking, alcohol, family history of myocardial infarction, BMI, vitamin supplement use, vitamin E use, physical activity, aspirin use, 2-year follow-tip period, presence of hypertension, presence of hypercholesterolemi a, energy intake, and postmenopausal hormone use	14	0.78 (NA)<br><br>5 vs. 1 quintile
Joshipura	2001	USA	HPFS	40-75	M	*42,148*	*1,063*	FFQ, validated	CHD	Records were reviewed by physicians who were blinded to the participants' risk factor status.	Potatoes	Age, smoking, alcohol, family history of myocardial infarction, BMI, vitamin supplement use, vitamin E use, physical activity, aspirin use, 2-year follow-tip period, presence of hypertension, presence of hypercholesterolemi a., energy intake	8	1.41 (NA)<br><br>5 vs. 1 quintile
Kahn	1984	US	AHS	>30	Both	*27,530*	*247*	Dietary questionnaire	All-cause mortality	Death tapes	Potatoes	NA	21	0.81 (0.67, 0.97)<br><br>4 vs. 1 quartile
Kato	1997	US	NYUWH S	34-65	W	*14,727*	*100*	FFQ	CRC	Regional cancer registries	Potatoes	Age, energy intake and place at enrolment and highest level of education	7.1	1.05 (0.62, 1.79)<br><br>4 vs. 1 quartile
Larsson	2016	Sweden	SMC	49-83	W	*32,805*	*MI: 1,437*	FFQ, validated	MI<br>Stroke	Linkage with the	Total potato consumption	Age, education, family history of MI	13	MI:<br>0.97 (0.80, 1.17)

							Stroke: 2,022<br><br>*HF: 1,177*		HF	Swedish National Patient Register and the Swedish Cause of Death Register	(Boiled potatoes, fried potatoes, and French fries)	before 60 y of age, smoking, aspirin use, walking or bicycling, exercise, BMI, history of hypertension, history of hypercholesterolemia, alcohol consumption, energy intake, mDASH diet score		Stroke:<br>0.86 (0.73, 1.00)<br><br>HF:<br>1.01 (0.82, 1.25)<br><br>5 vs. 1 quintile
Larsson	2016	Sweden	COSM	45-79	M	*36,508*	*MI: 2,979*<br><br>*Stroke: 2,509*<br><br>*HF: 1,462*	FFQ, validated	MI<br>Stroke<br>HF	Linkage with the Swedish National Patient Register and the Swedish Cause of Death Register	Total potato consumption (Boiled potatoes, fried potatoes, and French fries)	Age, education, family history of MI before 60 y of age, smoking, aspirin use, walking or bicycling, exercise, BMI, history of hypertension, history of hypercholesterolemia, alcohol consumption, energy intake, mDASH diet score	13	MI:<br>1.05 (0.92, 1.19)<br><br>Stroke:<br>1.00 (0.87, 1.15)<br><br>HF:<br>0.91 (0.75, 1.10)<br><br>5 vs. 1 quintile
Leenders	2013	Europe	EPIC	25-70	Both	*451,151*	*25,682*	Country-specific instruments	All-cause mortality	National Registry	Potatoes	Smoking, alcohol, BMI, physical activity, education, and processed meat consumption, fruit	12.8	1.00 (0.99, 1.01)<br><br>3 vs. 1 tertile
Liu	2004	USA	WHS	≥45	W	*38,018*	*1,614*	FFQ	T2D	Self-reported/ADA criteria	Potatoes and other tubers	Age, energy intake, BMI, smoking, alcohol, physical activity, hypertension, hypercholesterolemia, family history of diabetes	8.8	1.02 (0.86, 1.22)<br><br>5 vs. 1 quintile
Michels	2000	US	NHS	30-55	W	*88,764*	*724*	FFQ, validated	C, R	Family reports, National death Index, US	Total potato consumption (mashed or baked, French	Age, family history of colorectal cancer, sigmoidoscopy, height, BMI, pack-	6	C:<br>1.12 (NA)<br><br>R:

										Postal Service, medical and pathology reports	fries, potato chips)	years of smoking, alcohol intake, physical activity, aspirin use, vitamin supplement intake, total caloric intake, and red meat consumption.		1.03 (0.86, 1.22)<br><br>5 vs. 1 quintile
Michels	2000	US	HPFS	40-75	M	*47,325*	*457*	FFQ, validated	C, R	Family reports, National death Index , US Postal Service, medical and pathology reports	Total potato consumption (mashed or baked, French fries, potato chips)	Age, family history of colorectal cancer, sigmoidoscopy, height, BMI, pack-years of smoking, alcohol intake, physical activity, aspirin use, vitamin supplement intake, total caloric intake, and red meat consumption.	6	C:<br>1.07 (NA)<br><br>R:<br>1.83 (0.86, 1.22)<br><br>5 vs. 1 quintile
Mizrahi	2009	Finland	FMCHES	40-74	Both	*3,932*	*625*	1-year dietary history interview	Stroke	Finnish Hospital Discharge Register; Statistics Finland	Potatoes	Age, sex, BMI, smoking, physical activity, serum cholesterol level, blood pressure and energy intake	24	0.86 (0.68, 1.09)<br><br>4 vs. 1 quartile
Montonen	2003	Finland	FMCHES	40-69	Both	*4,316*	*156*	Dietary history interview	T2D	Registration to the Social Insurance Institution	Potatoes	Age, sex, area, energy intake, BMI, smoking, vegetables, fruit and berries	10	1.42 (1.02, 1.98)<br><br>4 vs. 1 quartile
Mucci	2006	Sweden	SMC	49-83	W	*61,467*	*741*	FFQ, validated	CRC	Swedish Cancer registry	Pan-fried potatoes	Age, BMI, education, alcohol, energy intake, saturated fat, fiber	823,072 person-years	1.10 (0.90, 1.40)<br><br>3 vs. 1 tertile
Muraki	2015	US	NHS	30-55	W	*70,773*	*7,436*	FFQ, validated	T2D	Self-reported (validated)	Total potato consumption (baked, boiled, or mashed potatoes and French fries)	Age, ethnicity smoking, alcohol intake, multivitamin use, physical activity,<br>a family history of diabetes, menopausal status	1,584,572 person-years	1.27 (1.04, 1.56)<br><br>5 vs. 1 quintile

											and postmenopausal hormone use, total energy intake, aHEI score, BMI		
Muraki	2015	US	NHS II	25-42	W	*87,739*	*4,621*	FFQ, validated	T2D	Self-reported (validated)	Total potato consumption (baked, boiled, or mashed potatoes and French fries)	Age, ethnicity smoking, alcohol intake, multivitamin use, physical activity, a family history of diabetes, menopausal status and postmenopausal hormone use, oral contraceptive use, total energy intake, aHEI score, BMI	1,610,311 person-years	1.38 (1.08, 1.76)<br><br>5 vs. 1 quintile
Muraki	2015	US	HPFS	40-75	M	*40,669*	*3,305*	FFQ, validated	T2D	Self-reported (validated)	Total potato consumption (baked, boiled, or mashed potatoes and French fries)	Age, ethnicity smoking, alcohol intake, multivitamin use, physical activity, a family history of diabetes, total energy intake, aHEI score, BMI	793,124 person-years	1.38 (1.07, 1.78)<br><br>5 vs. 1 quintile
Osella	2018	Italy	NUTRIHEP and MICOL	18-95<br><br>and >30	Both	*2,442*	*396*	FFQ, validated	All-cause mortality	Death certificates	Total potato consumption	Age, project, sex, job, educational level, systolic and diastolic blood pressure at baseline, adherence to Mediterranean diet, BMI, presence of any disease, alcohol intake, total energy intake	11	0.75 (0.53, 1.07)<br><br>5 vs. 1 quintile
Sonested	2015	Sweden	MDCS	44-74	Both	*26,445*	*1,344/ 1,176*	Modified diet history method, FFQ, validated	CHD<br><br>Stroke	Linkage with Swedish Hospital Discharge Registry and Cause-of-	Potatoes	Age, sex, season, diet method version, energy intake, BMI, smoking, alcohol consumption, leisure-time	14	CHD:<br>1.01 (0.86, 1.17)<br><br>Stroke:<br>1.08 (0.90, 1.31)

										death Registry		physical activity, and education.		5 vs. 1 quintile
Steinmetz	1994	US	IWHS	55-69	W	*41,837*	*212*	FFQ	C	State Health Registry of Iowa	Potatoes	Age, energy intake	5	1.24 (0.76, 2.04)<br><br>5 vs. 1 quartile
Trichopoulou	2005	Greece	EPIC-Greece	20-86	Both	*1,302*	*131*	FFQ, validated	All-cause mortality	Death certificates, which were available in all instances	Potatoes	Age, sex, previous treatment for hypertension and/or previous treatment for hypercholesterolemia, diabetes mellitus at enrolment, years of schooling, smoking status, waist-to-hip ratio, metabolic equivalent score, body mass index, energy intake	3.8	0.97 (0.80, 1.18)<br><br>per 40 g/d increment
Veronese	2017	US	OICS	45-79	Both	*4,440*	*236*	FFQ, validated	All-cause mortality	Confirmed and adjudicated by several methods (autopsy report, coroner's report, death certificate medical records, National Death Index, obituary, or Social Security Death Index)	Fried potatoes	Age, sex, race/ ethnicity, BMI, education, smoking habits, yearly income, Physical Activity Scale for Elderly score, Charlson comorbidity index, daily energy intake, alcohol consumption, adherence to a Mediterranean diet, and Centre for Epidemiologic Studies-Depression scale	8	1.11 (0.65, 1.91)<br><br>5 vs. 1 quartile
Villegas	2007	China	SWHS	40-70	W	*64,227*	*1,608*	FFQ, validated	T2D	Identified through outcome follow-	Potatoes; Sweet potatoes	Multivariable adjusted	4.6	Potatoes<br>0.72 (NA)<br><br>Sweet potatoes

										up surveys				0.72 (0.51)<br><br>5 vs. 1 quintile
von Ruesten	2013	Germany	EPIC-Potsdam	35-65	Both	*23,531*	*837*	FFQ, validated	T2D	Self-reported /inquiry to the physician	Potatoes; Fried potatoes	Age, sex, energy intake, Body Mass Index, waist: hip ratio, smoking, alcohol, physical activity, Hypertension, high blood lipid levels, education, vitamin supplementation	8	Potatoes:<br>1.10 (0.95, 1.27)<br><br>per 100 g/d increase<br><br>Fried potatoes:<br>1.21 (0.75, 1.97)<br><br>per 100 g/d increase
Würtz	2016	Denmark	DDCHS	50–64	Both	*55,171*	*2,350*	FFQ, validated	MI	Danish National Patient Register, Danish Cause of Death Register, Central Population Register	Potatoes	Age, energy intake, alcohol abstinence, alcohol intake, BMI, waist circumference, smoking status, physical activity, duration of schooling, menopausal status and use of hormone replacement therapy, fruits, sweets, soft drinks, lean dairy products, fatty dairy products, potato chips, refined cereals, wholegrain cereals, nuts	13.6	1.03 (0.91, 1.17)<br><br>Per 150 g/d
Ye	2006	Taiwan	7TT	≥30	Both	*22,115*	*107*	FFQ	CRC	Reviewing medical records and death certificates	Sweet Potatoes	NA	10	1.85 (1.08, 3.17)<br><br>Highest vs. lowest category

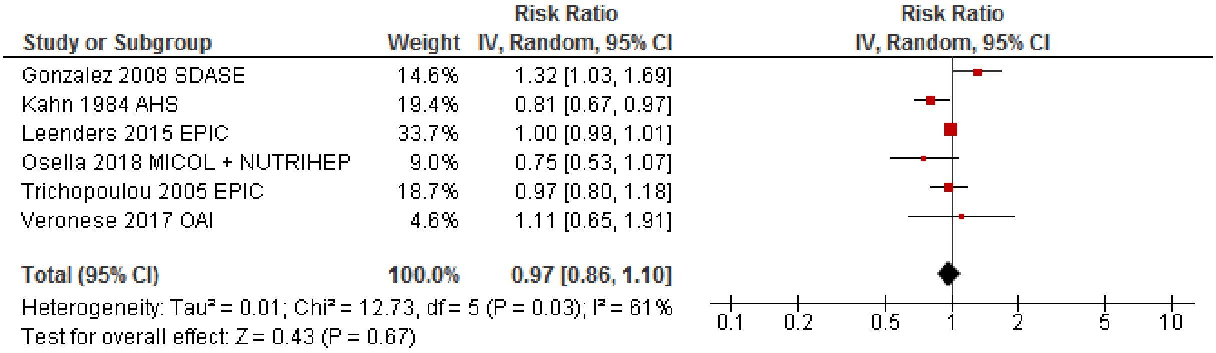

ESM Figure 2: Summary of relative risk of all-cause mortality for high versus low potatoes intake. 95% CI, 95% confidence interval.

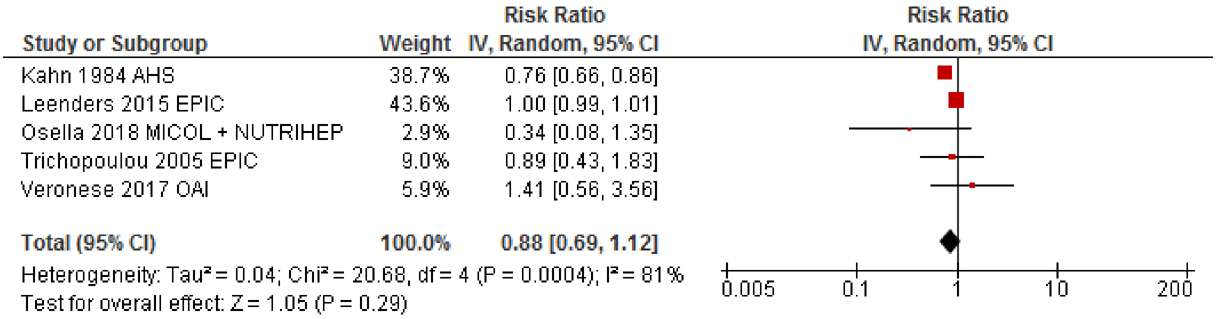

ESM Figure 3: Summary of relative risk of all-cause mortality for each 150 g/d increase in potatoes intake. 95% CI, 95% confidence interval.

Study or Subgroup	Weight	Risk Ratio IV, Random, 95% CI
**1.2.1 CHD**		
Dilis 2012 EPIC-Greece	41.1%	1.01 [0.91, 1.12]
Joshipura 2001 NHS + HPFS	1.0%	1.15 [0.59, 2.24]
Larsson 2016 COSM	26.8%	1.05 [0.92, 1.19]
Larsson 2016 SMC	12.5%	0.97 [0.80, 1.17]
Sönestedt 2015 MDCS	18.6%	1.01 [0.86, 1.18]
**Subtotal (95% CI)**	**100.0%**	**1.02 [0.95, 1.09]**
Heterogeneity: Tau² = 0.00; Chi² = 0.63, df = 4 (P = 0.96); I² = 0%		
Test for overall effect: Z = 0.49 (P = 0.63)		
**1.2.2 stroke**		
Joshipura 1999 HPFS	7.9%	1.23 [0.88, 1.72]
Joshipura 1999 NHS	4.5%	1.09 [0.69, 1.72]
Larsson 2016 COSM	28.6%	1.00 [0.87, 1.15]
Larsson 2016 SMC	24.9%	0.86 [0.73, 1.01]
Mizrahi 2009 FMCHES	14.3%	0.86 [0.68, 1.09]
Sönestedt 2015 MDCS	19.8%	1.08 [0.90, 1.30]
**Subtotal (95% CI)**	**100.0%**	**0.98 [0.88, 1.08]**
Heterogeneity: Tau² = 0.00; Chi² = 6.88, df = 5 (P = 0.23); I² = 27%		
Test for overall effect: Z = 0.46 (P = 0.65)		

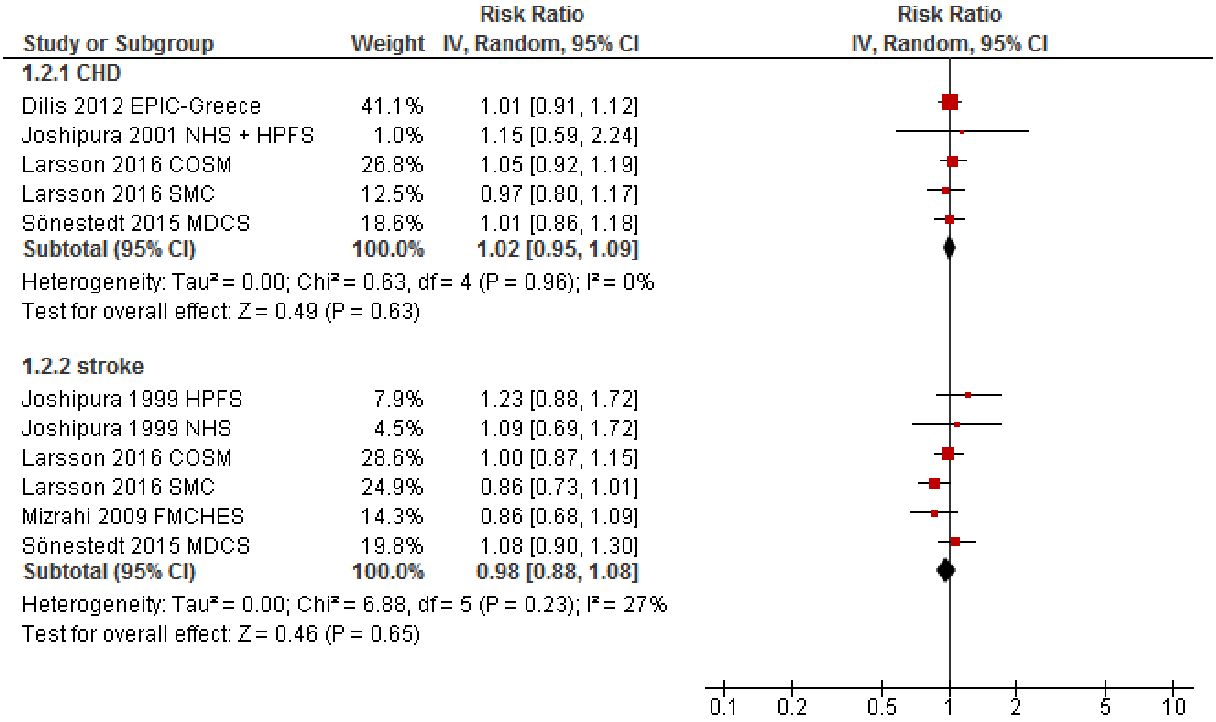

ESM Figure 4: Summary of relative risk of coronary heart disease and stroke for high versus low potatoes intake. 95% CI, 95% confidence interval.

Study or Subgroup	Weight	Risk Ratio IV, Random, 95% CI
**2.2.1 CHD**		
Dilis 2012 EPIC-Greece	6.1%	1.03 [0.80, 1.33]
Joshipura 2001 NHS + HPFS	1.2%	1.06 [0.59, 1.89]
Larsson 2016 COSM	31.9%	1.05 [0.94, 1.17]
Larsson 2016 SMC	15.7%	1.00 [0.85, 1.17]
Sönestedt 2015 MDCS	20.0%	1.00 [0.87, 1.15]
Würtz 2016 DDCS	25.2%	1.03 [0.91, 1.17]
**Subtotal (95% CI)**	**100.0%**	**1.03 [0.96, 1.09]**
Heterogeneity: Tau² = 0.00; Chi² = 0.47, df = 5 (P = 0.99); I² = 0%		
Test for overall effect: Z = 0.81 (P = 0.42)		
**2.2.2 stroke**		
Joshipura 1999 HPFS	1.1%	1.15 [0.69, 1.90]
Joshipura 1999 NHS	2.0%	1.25 [0.85, 1.83]
Larsson 2016 COSM	19.6%	1.01 [0.90, 1.14]
Larsson 2016 SMC	16.7%	0.91 [0.80, 1.04]
Mizrahi 2009 FMCHES	43.5%	0.95 [0.88, 1.03]
Sönestedt 2015 MDCS	17.1%	1.05 [0.92, 1.19]
**Subtotal (95% CI)**	**100.0%**	**0.98 [0.93, 1.03]**
Heterogeneity: Tau² = 0.00; Chi² = 5.16, df = 5 (P = 0.40); I² = 3%		
Test for overall effect: Z = 0.81 (P = 0.42)		

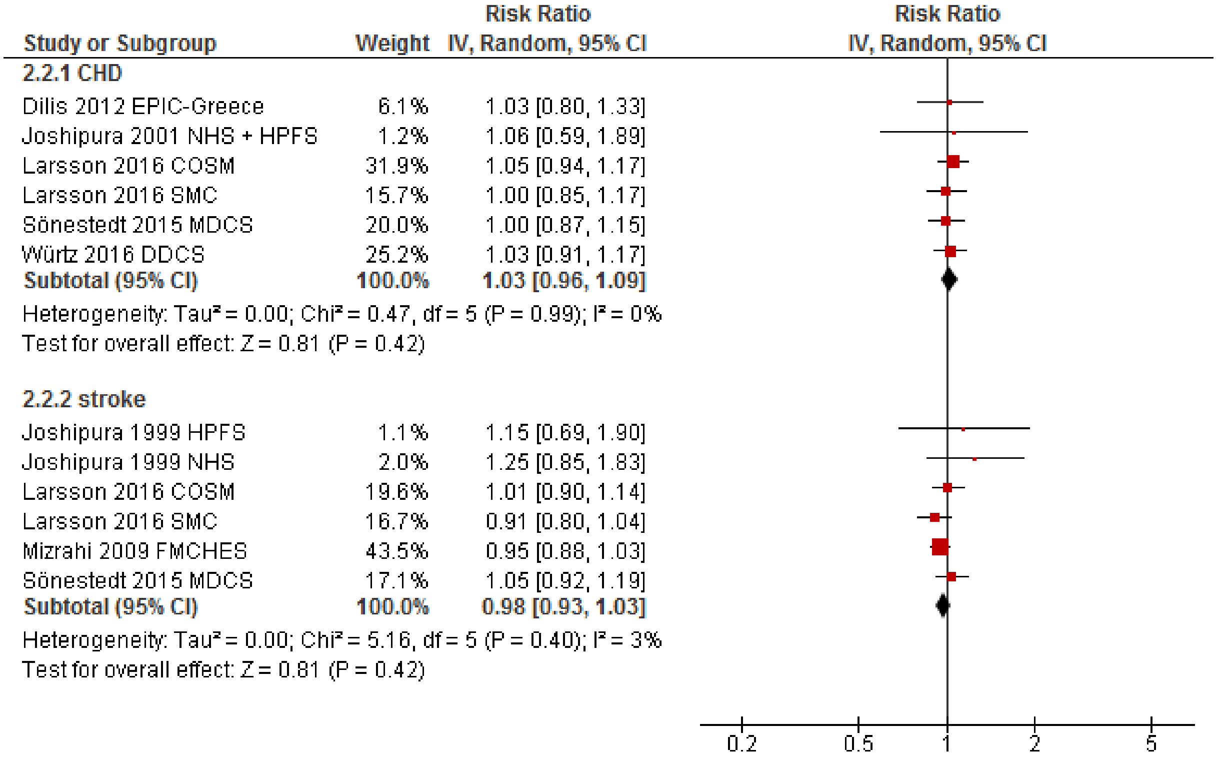

ESM Figure 5: Summary of relative risk of coronary heart disease and stroke for each 150 g/d increase in potatoes intake. 95% CI, 95% confidence interval.

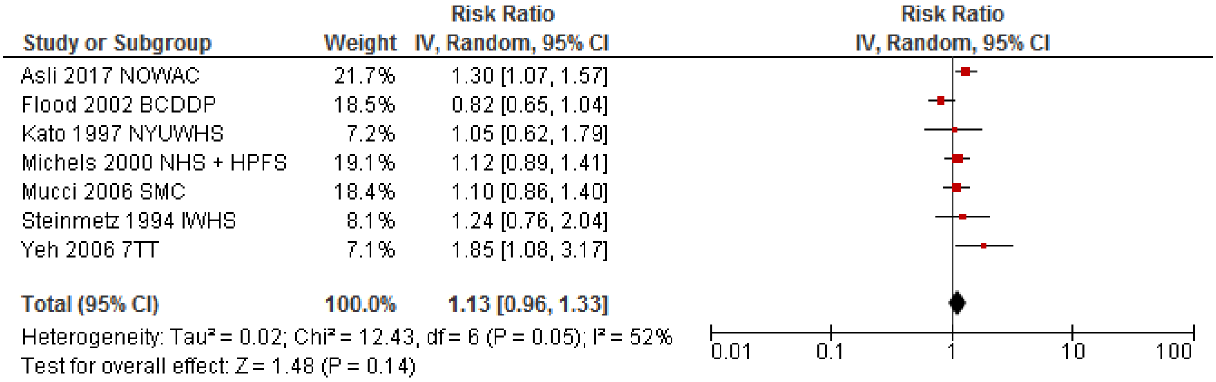

ESM Figure 6: Summary of relative risk of colorectal cancer for high versus low potatoes intake. 95% CI, 95% confidence interval.

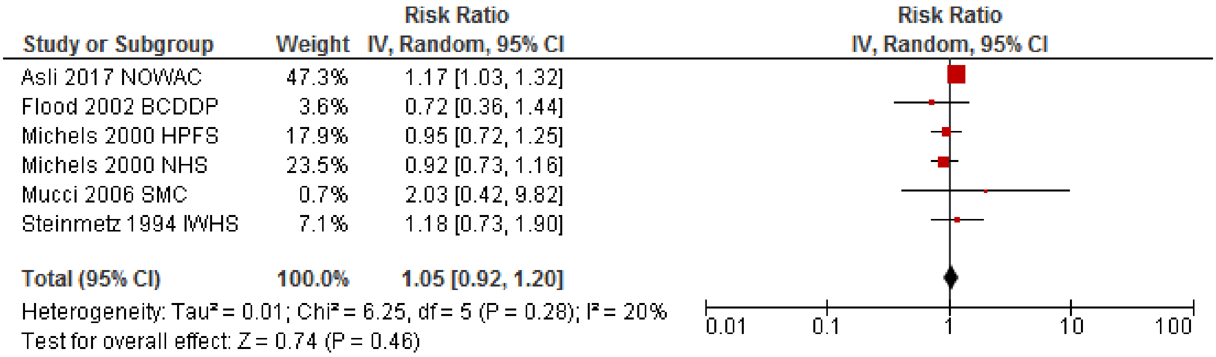

ESM Figure 7: Summary of relative risk of colorectal cancer for each 150 g/d increase in potatoes intake. 95% CI, 95% confidence interval.

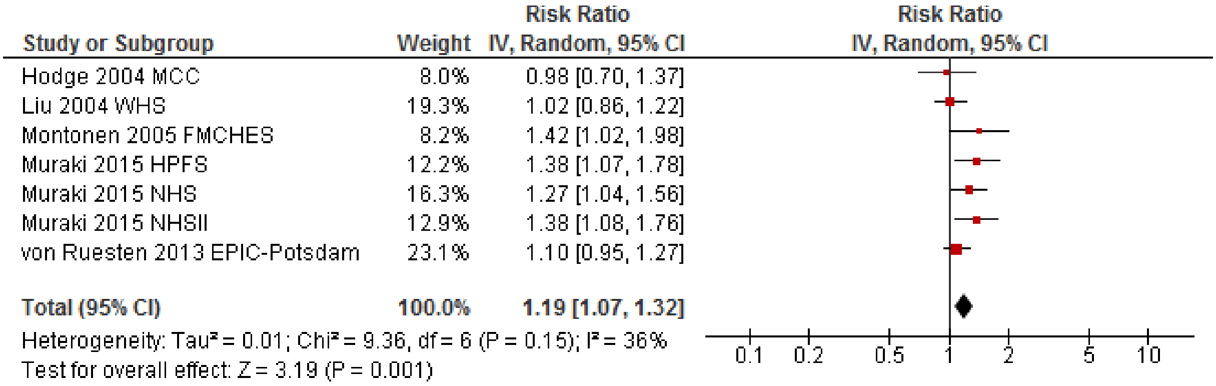

ESM Figure 8: Summary of relative risk of type 2 diabetes for high versus low potatoes intake. 95% CI, 95% confidence interval.

Study or Subgroup	Weight	Risk Ratio IV, Random, 95% CI
Hodge 2004 MCC	5.4%	0.95 [0.71, 1.27]
Liu 2004 WHS	12.4%	1.00 [0.84, 1.19]
Montonen 2005 FMCHES	16.2%	1.18 [1.02, 1.36]
Muraki 2015 HPFS	16.3%	1.28 [1.10, 1.48]
Muraki 2015 NHS	23.2%	1.22 [1.09, 1.35]
Muraki 2015 NHSII	17.1%	1.29 [1.12, 1.48]
von Ruesten 2013 EPIC-Potsdam	9.3%	1.15 [0.93, 1.42]
**Total (95% CI)**	**100.0%**	**1.18 [1.10, 1.27]**

Heterogeneity: Tau² = 0.00; Chi² = 8.58, df = 6 (P = 0.20); I² = 30%
Test for overall effect: Z = 4.45 (P < 0.00001)

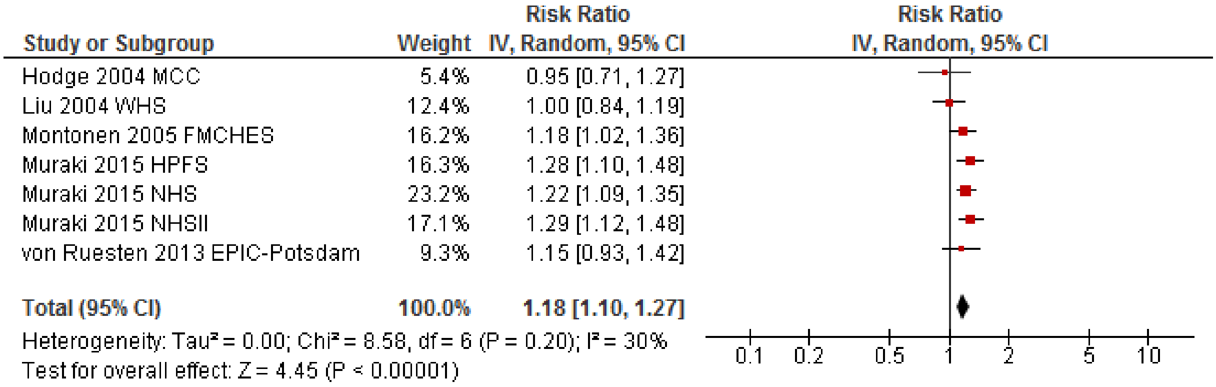

ESM Figure 9: Summary of relative risk of type 2 diabetes for each 150 g/d increase in potatoes intake. 95% CI, 95% confidence interval.

Study or Subgroup	Weight	Risk Ratio IV, Random, 95% CI
**3.2.1 French fries**		
Muraki 2015 NHS + NHS II + HPFS	95.5%	1.68 [1.44, 1.96]
von Ruesten 2013 EPIC-Potsdam	4.5%	1.33 [0.65, 2.72]
**Subtotal (95% CI)**	**100.0%**	**1.66 [1.43, 1.94]**
Heterogeneity: Tau² = 0.00; Chi² = 0.39, df = 1 (P = 0.53); I² = 0%		
Test for overall effect: Z = 6.53 (P < 0.00001)		
**3.2.2 Baked/boiled/mashed potatoes**		
Muraki 2015 NHS + NHS II + HPFS	87.8%	1.08 [1.00, 1.18]
von Ruesten 2013 EPIC-Potsdam	12.2%	1.15 [0.93, 1.43]
**Subtotal (95% CI)**	**100.0%**	**1.09 [1.01, 1.18]**
Heterogeneity: Tau² = 0.00; Chi² = 0.24, df = 1 (P = 0.62); I² = 0%		
Test for overall effect: Z = 2.28 (P = 0.02)		

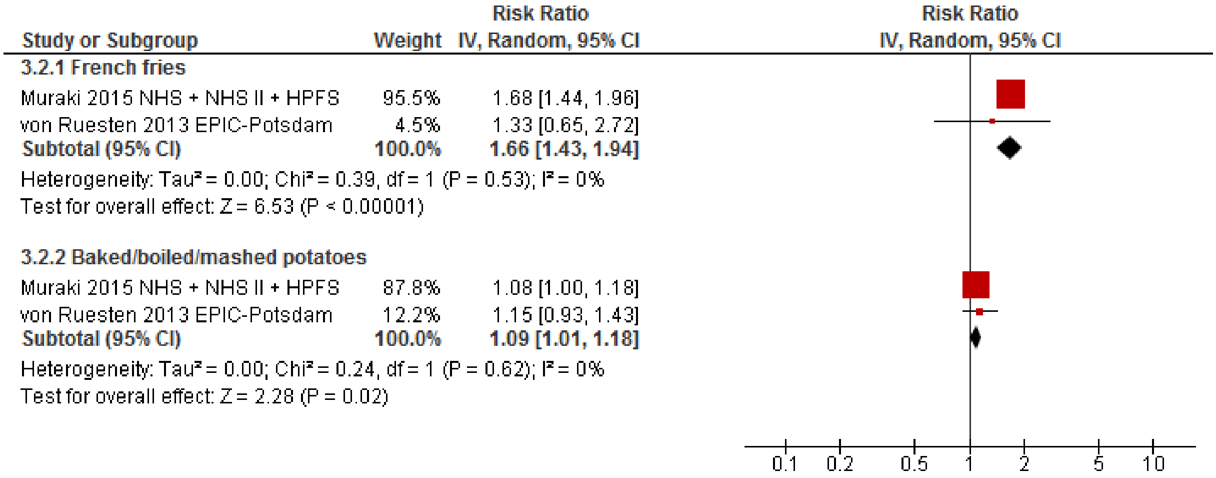

Test for subgroup differences: Chi² = 23.26, df = 1 (P < 0.00001), I² = 95.7%

ESM Figure 10: Summary of relative risk of type 2 diabetes for each 150 g/d increase in French fries vs. Baked/ boiled/ mashed potatoes intake. 95% CI, 95% confidence interval.

Study or Subgroup	Weight	Risk Ratio IV, Random, 95% CI
Borgi 2016 HPFS	24.3%	0.96 [0.79, 1.16]
Borgi 2016 NHS	27.5%	1.07 [0.92, 1.24]
Borgi 2016 NHSII	24.4%	1.42 [1.18, 1.72]
Hu 2017 SUN	23.7%	0.98 [0.80, 1.19]
**Total (95% CI)**	**100.0%**	**1.09 [0.92, 1.29]**

Heterogeneity: Tau² = 0.02; Chi² = 10.37, df = 3 (P = 0.02); I² = 71%
Test for overall effect: Z = 1.04 (P = 0.30)

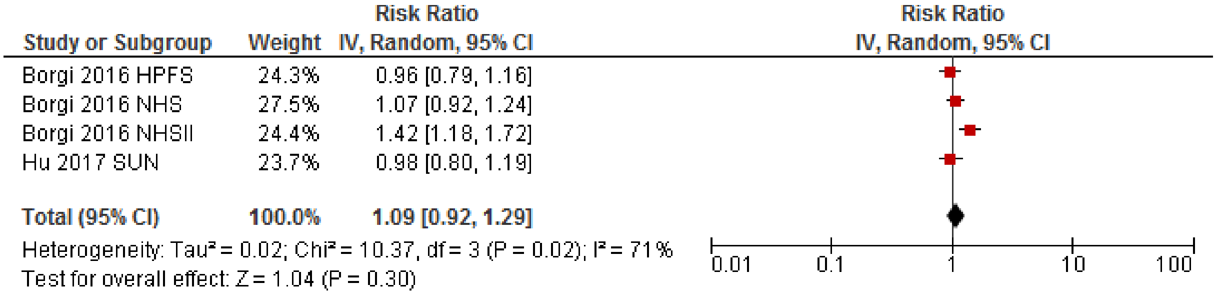

ESM Figure 11: Summary of relative risk of hypertension for high versus low potatoes intake. 95% CI, 95% confidence interval.

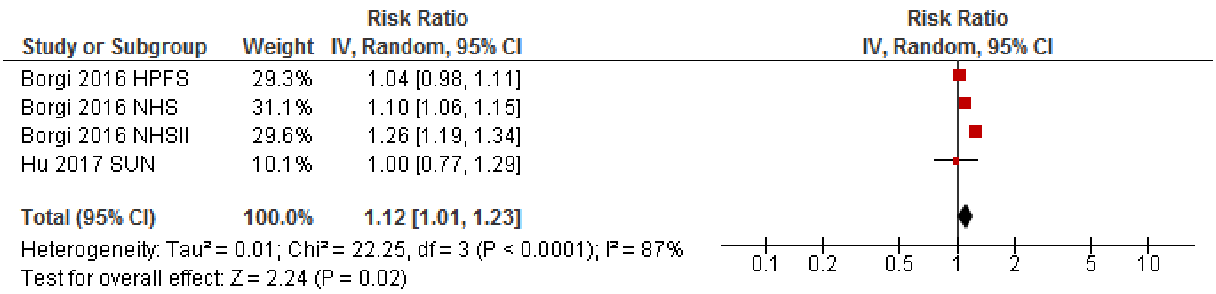

ESM Figure 12: Summary of relative risk of hypertension for each 150 g/d increase in potatoes intake. 95% CI, 95% confidence interval.

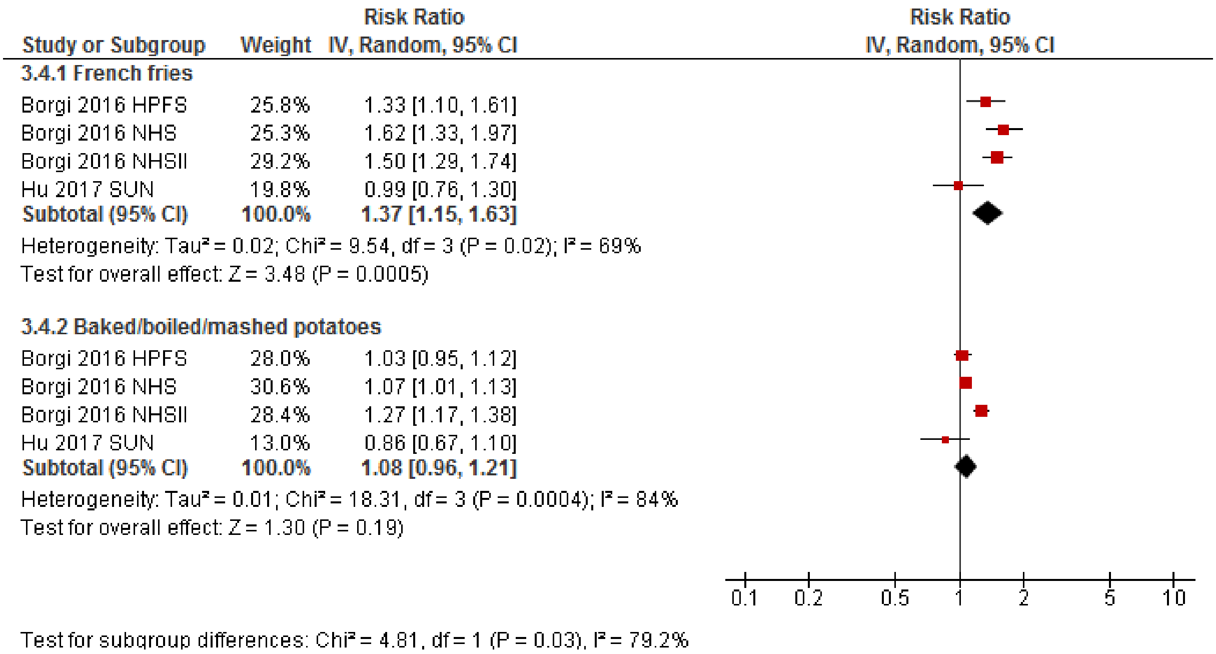

ESM Figure 13: Summary of relative risk of hypertension for each 150 g/d increase in French fries vs. Baked/ boiled/ mashed potatoes intake. 95% CI, 95% confidence interval.

ESM Table 2. Dose-response meta-analysis for each daily 150 gram increase in potatoes intake and risk of CHD, stratified by sex, follow-up, geographic location, and number of cases, and dietary assessment

CHD	No of studies	RR	95% CI	I^2 (%)	p-value for subgroup difference
Dose-response	7	1.03	0.96, 1.09	0	
Sex					
Men and women	5	1.02	0.94, 1.11	0	
Men	1	1.05	0.94, 1.17	NA	0.84
Women	1	1.00	0.85, 1.17	NA	
Follow-up					
≥10 years	7	1.03	0.96, 1.09	0	
Geographic location					
Europe	5	1.03	0,96, 1.09	0	0.91
America	2	1.06	0.59, 1.89	NA	
Number of cases					
≥1000	4	1.03	0.96, 1.09	0	0.94
<1000	3	1.03	0.82, 1.30	0	
Dietary assessment					
validated	7	1.03	0.96, 1.09	0	

ESM Table 3. Dose-response meta-analysis for each daily 150 gram increase in potatoes intake and risk of stroke, stratified by sex, follow-up, geographic location, and number of cases, and dietary assessment

Stroke	No of studies	RR	95% CI	I^2 (%)	p-value for subgroup difference
Dose-response	6	0.98	0.93, 1.03	3	
Sex					
Men and women	2	0.98	0.90, 1.08	39	
Men	2	1.02	0.91, 1.15	0	0.95
Women	2	1.01	0.75, 1.36	58	
Follow-up					
≥10 years	6	0.98	0.93, 1.03	3	
Geographic location					
Europe	4	0.97	0.92, 1.03	3	0.16
America	2	1.21	0.89, 1.64	0	
Number of cases					
≥1000	3	0.99	0.91, 1.07	19	0.94
<1000	3	1.00	0.86, 1.15	17	
Dietary assessment					
validated	5	1.00	0.93, 1.08	4	0.32
not validated	1	0.95	0.88, 1.03	NA	

ESM Table 4. Dose-response meta-analysis for each daily 150 gram increase in potatoes intake and risk of CRC, stratified by sex, follow-up, geographic location, and number of cases, and dietary assessment

CRC	No of studies	RR	95% CI	I^2 (%)	p-value for subgroup difference
Dose-response	6	1.05	0.92, 1.20	20	
Sex					
Men	1	0.95	0.72, 1.25	NA	0.47
Women	5	1.07	0.91, 1.25	24	
Follow-up					
<10 years	4	0.94	0.80, 1.11	0	0.04
≥10 years	2	1.17	1.04, 1.33	0	
Geographic location					
Europe	2	1.17	1.04, 1.33	0	0.04
America	4	0.94	0.80, 1.11	0	
Number of cases					
<1000	6	1.05	0.92, 1.20	20	
Dietary assessment					
validated	5	1.00	0.93, 1.08	4	0.32
not validated	1	0.95	0.88, 1.03	NA	

ESM Table 5. Dose-response meta-analysis for each daily 150 gram increase in potatoes intake and risk of type 2 diabetes, stratified by sex, follow-up, geographic location, and number of cases, and dietary assessment

Type 2 diabetes	No of studies	RR	95% CI	I² (%)	p-value for subgroup difference
Dose-response	7	1.18	1.10, 1.27	30	
Sex					
Men and women	3	1.13	1.01, 1.27	0	
Men	1	1.28	1.10, 1.48	NA	0.44
Women	3	1.17	1.03, 1.34	61	
Follow-up					
≥10 years	4	1.23	1.16, 1.32	0	0.01
<10 years	3	1.04	0.92, 1.17	0	
Geographic location					
Europe	3	1.11	1.00, 1.23	6	0.05
America	3	1.25	1.16, 1.35	0	
Asia & Australia	1	0.95	0.71, 1.27	NA	
Number of cases					
≥1000	6	1.20	1.12, 1.28	18	0.12
<1000	1	0.95	0.71, 1.27	NA	
Dietary assessment					
validated	5	1.03	0.88, 1.21	35	0.59
not validated	1	1.18	0.73, 1.90	NA	

ESM Table 6: Linear dose-response meta-analysis investigating the association between potatoes intake and the risk of all-cause mortality, coronary heart disease, stroke, colorectal cancer, type 2 diabetes and hypertension, and NutriGrade grading.

Outcome	Type of potato consumption	No of studies	RR	95% CI	I^2 (%)	NutriGrade grading
All-cause mortality	Total potato consumption	5	0.88	0.69, 1.12	81	Low
Coronary heart disease	Total potato consumption	7	1.03	0.96, 1.09	0	Low
Stroke	Total potato consumption	6	0.98	0.93, 1.03	3	Low
Colorectal cancer	Total potato consumption	6	1.05	0.92, 1.20	20	Low
Type 2 diabetes	Total potato consumption	7	1.18	1.10, 1.27	30	Low
	French Fries	4	1.66	1.43, 1.94	0	Moderate
	Baked/ boiled/ mashed	4	1.09	1.01, 1.18	0	Low
Hypertension	Total potato consumption	4	1.12	1.01, 1.23	87	Low
	French Fries	4	1.37	1.15, 1.63	69	Moderate
	Baked/ boiled/ mashed	4	1.08	0.96, 1.21	84	Low

ESM References

1. Bazzano LA, Li TY, Joshipura KJ, Hu FB. Intake of fruit, vegetables, and fruit juices and risk of diabetes in women. Diabetes Care 2008;31(7):1311-7. doi: 10.2337/dc08-0080.

2. Belin RJ, Greenland P, Allison M, Martin L, Shikany JM, Larson J, Tinker L, Howard BV, Lloyd-Jones D, Van Horn L. Diet quality and the risk of cardiovascular disease: the Women's Health Initiative (WHI). Am J Clin Nutr 2011;94(1):49-57. doi: 10.3945/ajcn.110.011221.

3. Bendinelli B, Masala G, Saieva C, Salvini S, Calonico C, Sacerdote C, Agnoli C, Grioni S, Frasca G, Mattiello A, et al. Fruit, vegetables, and olive oil and risk of coronary heart disease in Italian women: the EPICOR Study. Am J Clin Nutr 2011;93(2):275-83. doi: 10.3945/ajcn.110.000521.

4. Bhupathiraju SN, Wedick NM, Pan A, Manson JE, Rexrode KM, Willett WC, Rimm EB, Hu FB. Quantity and variety in fruit and vegetable intake and risk of coronary heart disease. Am J Clin Nutr 2013;98(6):1514-23. doi: 10.3945/ajcn.113.066381.

5. Buckland G, Gonzalez CA, Agudo A, Vilardell M, Berenguer A, Amiano P, Ardanaz E, Arriola L, Barricarte A, Basterretxea M, et al. Adherence to the Mediterranean diet and risk of coronary heart disease in the Spanish EPIC Cohort Study. Am J Epidemiol 2009;170(12):1518-29. doi: 10.1093/aje/kwp282.

6. Cooper AJ, Forouhi NG, Ye Z, Buijsse B, Arriola L, Balkau B, Barricarte A, Beulens JW, Boeing H, Buchner FL, et al. Fruit and vegetable intake and type 2 diabetes: EPIC-InterAct prospective study and meta-analysis. Eur J Clin Nutr 2012;66(10):1082-92. doi: 10.1038/ejcn.2012.85.

7. Dauchet L, Amouyel P, Hercberg S, Dallongeville J. Fruit and vegetable consumption and risk of coronary heart disease: a meta-analysis of cohort studies. J Nutr 2006;136(10):2588-93.

8. Del Gobbo LC, Kalantarian S, Imamura F, Lemaitre R, Siscovick DS, Psaty BM, Mozaffarian D. Contribution of Major Lifestyle Risk Factors for Incident Heart Failure in Older Adults: The Cardiovascular Health Study. JACC Heart failure 2015;3(7):520-8. doi: 10.1016/j.jchf.2015.02.009.

9. Ford ES, Bergmann MM, Kroger J, Schienkiewitz A, Weikert C, Boeing H. Healthy living is the best revenge: findings from the European Prospective Investigation Into Cancer and Nutrition-Potsdam study. Arch Intern Med 2009;169(15):1355-62. doi: 10.1001/archinternmed.2009.237.

10. Fraser GE, Shavlik DJ. Risk factors for all-cause and coronary heart disease mortality in the oldest-old. The Adventist Health Study. Arch Intern Med 1997;157. doi: 10.1001/archinte.1997.00440400099012.

11. Fraser GE, Sumbureru D, Pribis P, Neil RL, Frankson MA. Association among health habits, risk factors, and all-cause mortality in a black California population. Epidemiology (Cambridge, Mass) 1997;8(2):168-74.

12. Genkinger JM, Platz EA, Hoffman SC, Comstock GW, Helzlsouer KJ. Fruit, vegetable, and antioxidant intake and all-cause, cancer, and cardiovascular disease mortality in a community-dwelling population in Washington County, Maryland. Am J Epidemiol 2004;160(12):1223-33. doi: 10.1093/aje/.

13. Gillman MW, Cupples LA, Gagnon D, Posner BM, Ellison RC, Castelli WP, Wolf PA. Protective effect of fruits and vegetables on development of stroke in men. Jama 1995;273(14):1113-7.

14. Hansen CP, Overvad K, Kyro C, Olsen A, Tjonneland A, Johnsen SP, Jakobsen MU, Dahm CC. Adherence to a Healthy Nordic Diet and Risk of Stroke: A Danish Cohort Study. Stroke 2017;48(2):259-64. doi: 10.1161/strokeaha.116.015019.

15. Hansen L, Dragsted LO, Olsen A, Christensen J, Tjonneland A, Schmidt EB, Overvad K. Fruit and vegetable intake and risk of acute coronary syndrome. Br J Nutr 2010;104(2):248-55. doi: 10.1017/s0007114510000462.

16. Hirvonen T, Pietinen P, Virtanen M, Ovaskainen ML, Hakkinen S, Albanes D, Virtamo J. Intake of flavonols and flavones and risk of coronary heart disease in male smokers. Epidemiology (Cambridge, Mass) 2001;12(1):62-7.

17. Johnsen SP, Overvad K, Stripp C, Tjonneland A, Husted SE, Sorensen HT. Intake of fruit and vegetables and the risk of ischemic stroke in a cohort of Danish men and women. Am J Clin Nutr 2003;78(1):57-64.

18. Keli SO, Hertog MG, Feskens EJ, Kromhout D. Dietary flavonoids, antioxidant vitamins, and incidence of stroke: the Zutphen study. Arch Intern Med 1996;156(6):637-42.

19. Knoops KT, de Groot LC, Kromhout D, Perrin AE, Moreiras-Varela O, Menotti A, van Staveren WA. Mediterranean diet, lifestyle factors, and 10-year mortality in elderly European men and women: the HALE project. Jama 2004;292(12):1433-9. doi: 10.1001/jama.292.12.1433.

20. Kobylecki CJ, Afzal S, Davey Smith G, Nordestgaard BG. Genetically high plasma vitamin C, intake of fruit and vegetables, and risk of ischemic heart disease and all-cause mortality: a Mendelian randomization study. Am J Clin Nutr 2015;101(6):1135-43. doi: 10.3945/ajcn.114.104497.

21. Kurotani K, Nanri A, Goto A, Mizoue T, Noda M, Kato M, Inoue M, Tsugane S. Vegetable and fruit intake and risk of type 2 diabetes: Japan Public Health Center-based Prospective Study. Br J Nutr 2013;109(4):709-17. doi: 10.1017/s0007114512001705.

22. Lin J, Zhang SM, Cook NR, Rexrode KM, Liu S, Manson JE, Lee IM, Buring JE. Dietary intakes of fruit, vegetables, and fiber, and risk of colorectal cancer in a prospective cohort of women (United States). Cancer causes & control : CCC 2005;16(3):225-33. doi: 10.1007/s10552-004-4025-1.

23. Liu S, Lee IM, Ajani U, Cole SR, Buring JE, Manson JE. Intake of vegetables rich in carotenoids and risk of coronary heart disease in men: The Physicians' Health Study. Int J Epidemiol 2001;30(1):130-5.

24. Liu S, Manson JE, Lee IM, Cole SR, Hennekens CH, Willett WC, Buring JE. Fruit and vegetable intake and risk of cardiovascular disease: the Women's Health Study. Am J Clin Nutr 2000;72(4):922-8.

25. Mamluk L, O'Doherty MG, Orfanos P, Saitakis G, Woodside JV, Liao LM, Sinha R, Boffetta P, Trichopoulou A, Kee F. Fruit and vegetable intake and risk of incident of type 2 diabetes: results from the consortium on health and ageing network of cohorts in Europe and the United States (CHANCES). Eur J Clin Nutr 2016. doi: 10.1038/ejcn.2016.143.

26. Mann JI, Appleby PN, Key TJ, Thorogood M. Dietary determinants of ischaemic heart disease in health conscious individuals. Heart 1997;78(5):450-5.

27. Meyer KA, Kushi LH, Jacobs DR, Jr., Folsom AR. Dietary fat and incidence of type 2 diabetes in older Iowa women. Diabetes Care 2001;24(9):1528-35.

28. Misirli G, Benetou V, Lagiou P, Bamia C, Trichopoulos D, Trichopoulou A. Relation of the traditional Mediterranean diet to cerebrovascular disease in a Mediterranean population. Am J Epidemiol 2012;176(12):1185-92. doi: 10.1093/aje/kws205.

29. Mursu J, Virtanen JK, Tuomainen TP, Nurmi T, Voutilainen S. Intake of fruit, berries, and vegetables and risk of type 2 diabetes in Finnish men: the Kuopio Ischaemic Heart Disease Risk Factor Study. Am J Clin Nutr 2014;99(2):328-33. doi: 10.3945/ajcn.113.069641.

30. Nagura J, Iso H, Watanabe Y, Maruyama K, Date C, Toyoshima H, Yamamoto A, Kikuchi S, Koizumi A, Kondo T, et al. Fruit, vegetable and bean intake and mortality from cardiovascular disease among Japanese men and women: the JACC Study. Br J Nutr 2009;102(2):285-92. doi: 10.1017/s0007114508143586.

31. Neelakantan N, Naidoo N, Koh WP, Yuan JM, van Dam RM. The Alternative Healthy Eating Index Is Associated with a Lower Risk of Fatal and Nonfatal Acute Myocardial Infarction in a Chinese Adult Population. J Nutr 2016;146(7):1379-86. doi: 10.3945/jn.116.231605.

32. Nguyen B, Bauman A, Gale J, Banks E, Kritharides L, Ding D. Fruit and vegetable consumption and all-cause mortality: evidence from a large Australian cohort study. The international journal of behavioral nutrition and physical activity 2016;13:9. doi: 10.1186/s12966-016-0334-5.

33. Oude Griep LM, Geleijnse JM, Kromhout D, Ocke MC, Verschuren WM. Raw and processed fruit and vegetable consumption and 10-year coronary heart disease incidence in a population-based cohort study in the Netherlands. PLoS One 2010;5(10):e13609. doi: 10.1371/journal.pone.0013609.

34. Rautiainen S, Wang L, Lee IM, Manson JE, Buring JE, Sesso HD. Higher Intake of Fruit, but Not Vegetables or Fiber, at Baseline Is Associated with Lower Risk of Becoming Overweight or Obese in Middle-Aged and Older Women of Normal BMI at Baseline. J Nutr 2015;145(5):960-8. doi: 10.3945/jn.114.199158.

35. Shi Z, Zhang T, Byles J, Martin S, Avery JC, Taylor AW. Food Habits, Lifestyle Factors and Mortality among Oldest Old Chinese: The Chinese Longitudinal Healthy Longevity Survey (CLHLS). Nutrients 2015;7(9):7562-79. doi: 10.3390/nu7095353.

36. Stefler D, Malyutina S, Kubinova R, Pajak A, Peasey A, Pikhart H, Brunner EJ, Bobak M. Mediterranean diet score and total and cardiovascular mortality in Eastern Europe: the HAPIEE study. Eur J Nutr 2015. doi: 10.1007/s00394-015-1092-x.

37. Strandhagen E, Hansson PO, Bosaeus I, Isaksson B, Eriksson H. High fruit intake may reduce mortality among middle-aged and elderly men. The Study of Men Born in 1913. Eur J Clin Nutr 2000;54(4):337-41.

38. Tognon G, Nilsson LM, Lissner L, Johansson I, Hallmans G, Lindahl B, Winkvist A. The Mediterranean diet score and mortality are inversely associated in adults living in the subarctic region. J Nutr 2012;142(8):1547-53. doi: 10.3945/jn.112.160499.

39. Tognon G, Rothenberg E, Eiben G, Sundh V, Winkvist A, Lissner L. Does the Mediterranean diet predict longevity in the elderly? A Swedish perspective. Age (Dordrecht, Netherlands) 2011;33(3):439-50. doi: 10.1007/s11357-010-9193-1.

40. Tsubota-Utsugi M, Ohkubo T, Kikuya M, Metoki H, Kurimoto A, Suzuki K, Fukushima N, Hara A, Asayama K, Satoh H, et al. High fruit intake is associated with a lower risk of future hypertension determined by home blood pressure measurement: the OHASAMA study. Journal of human hypertension 2011;25(3):164-71. doi: 10.1038/jhh.2010.48.

41. Tucker KL, Hallfrisch J, Qiao N, Muller D, Andres R, Fleg JL. The combination of high fruit and vegetable and low saturated fat intakes is more protective against mortality in aging men than is either alone: the Baltimore Longitudinal Study of Aging. J Nutr 2005;135(3):556-61.

42. van den Brandt PA. The impact of a Mediterranean diet and healthy lifestyle on premature mortality in men and women. Am J Clin Nutr 2011;94(3):913-20. doi: 10.3945/ajcn.110.008250.

43. Villegas R, Shu XO, Gao YT, Yang G, Elasy T, Li H, Zheng W. Vegetable but not fruit consumption reduces the risk of type 2 diabetes in Chinese women. J Nutr 2008;138(3):574-80.

44. Wang JB, Fan JH, Dawsey SM, Sinha R, Freedman ND, Taylor PR, Qiao YL, Abnet CC. Dietary components and risk of total, cancer and cardiovascular disease mortality in the Linxian Nutrition Intervention Trials cohort in China. Sci Rep 2016;6:22619. doi: 10.1038/srep22619.

45. Wang L, Manson JE, Gaziano JM, Buring JE, Sesso HD. Fruit and vegetable intake and the risk of hypertension in middle-aged and older women. Am J Hypertens 2012;25(2):180-9. doi: 10.1038/ajh.2011.186.

46. Weng LC, Steffen LM, Szklo M, Nettleton J, Chambless L, Folsom AR. A diet pattern with more dairy and nuts, but less meat is related to lower risk of developing hypertension in middle-aged adults: the Atherosclerosis Risk in Communities (ARIC) study. Nutrients 2013;5(5):1719-33. doi: 10.3390/nu5051719.

47. Yu D, Zhang X, Gao YT, Li H, Yang G, Huang J, Zheng W, Xiang YB, Shu XO. Fruit and vegetable intake and risk of CHD: results from prospective cohort studies of Chinese adults in Shanghai. Br J Nutr 2014;111(2):353-62. doi: 10.1017/s0007114513002328.

48. Butler LM, Wang R, Koh WP, Yu MC. Prospective study of dietary patterns and colorectal cancer among Singapore Chinese. Br J Cancer 2008;99(9):1511-6. doi: 10.1038/sj.bjc.6604678.

49. Fung TT, Hu FB, Wu K, Chiuve SE, Fuchs CS, Giovannucci E. The Mediterranean and Dietary Approaches to Stop Hypertension (DASH) diets and colorectal cancer. Am J Clin Nutr 2010;92(6):1429-35. doi: 10.3945/ajcn.2010.29242.

50. George SM, Park Y, Leitzmann MF, Freedman ND, Dowling EC, Reedy J, Schatzkin A, Hollenbeck A, Subar AF. Fruit and vegetable intake and risk of cancer: a prospective cohort study. Am J Clin Nutr 2009;89(1):347-53. doi: 10.3945/ajcn.2008.26722.

51. Kunzmann AT, Coleman HG, Huang WY, Cantwell MM, Kitahara CM, Berndt SI. Fruit and vegetable intakes and risk of colorectal cancer and incident and recurrent adenomas in the PLCO cancer screening trial. Int J Cancer 2016;138(8):1851-61. doi: 10.1002/ijc.29922.

52. McCarl M, Harnack L, Limburg PJ, Anderson KE, Folsom AR. Incidence of colorectal cancer in relation to glycemic index and load in a cohort of women. Cancer epidemiology, biomarkers & prevention : a publication of the American Association for Cancer Research, cosponsored by the American Society of Preventive Oncology 2006;15(5):892-6. doi: 10.1158/1055-9965.epi-05-0700.

53. McCullough ML, Robertson AS, Chao A, Jacobs EJ, Stampfer MJ, Jacobs DR, Diver WR, Calle EE, Thun MJ. A prospective study of whole grains, fruits, vegetables and colon cancer risk. Cancer causes & control : CCC 2003;14(10):959-70.

54. Nomura AM, Wilkens LR, Murphy SP, Hankin JH, Henderson BE, Pike MC, Kolonel LN. Association of vegetable, fruit, and grain intakes with colorectal cancer: the Multiethnic Cohort Study. Am J Clin Nutr 2008;88(3):730-7.

55. Park Y, Subar AF, Kipnis V, Thompson FE, Mouw T, Hollenbeck A, Leitzmann MF, Schatzkin A. Fruit and vegetable intakes and risk of colorectal cancer in the NIH-AARP diet and health study. Am J Epidemiol 2007;166(2):170-80. doi: 10.1093/aje/kwm067.

56. Sanjoaquin MA, Appleby PN, Thorogood M, Mann JI, Key TJ. Nutrition, lifestyle and colorectal cancer incidence: a prospective investigation of 10998 vegetarians and non-vegetarians in the United Kingdom. Br J Cancer 2004;90(1):118-21. doi: 10.1038/sj.bjc.6601441.

57. Pietinen P, Malila N, Virtanen M, Hartman TJ, Tangrea JA, Albanes D, Virtamo J. Diet and risk of colorectal cancer in a cohort of Finnish men. Cancer causes & control : CCC 1999;10(5):387-96.

58. Sato Y, Tsubono Y, Nakaya N, Ogawa K, Kurashima K, Kuriyama S, Hozawa A, Nishino Y, Shibuya D, Tsuji I. Fruit and vegetable consumption and risk of colorectal cancer in Japan: The Miyagi Cohort Study. Public Health Nutr 2005;8(3):309-14.

59. Shibata A, Paganini-Hill A, Ross RK, Henderson BE. Intake of vegetables, fruits, beta-carotene, vitamin C and vitamin supplements and cancer incidence among the elderly: a prospective study. Br J Cancer 1992;66(4):673-9.

60. Singh PN, Fraser GE. Dietary risk factors for colon cancer in a low-risk population. Am J Epidemiol 1998;148(8):761-74.

61. Terry P, Giovannucci E, Michels KB, Bergkvist L, Hansen H, Holmberg L, Wolk A. Fruit, vegetables, dietary fiber, and risk of colorectal cancer. J Natl Cancer Inst 2001;93(7):525-33.

62. Tsubono Y, Otani T, Kobayashi M, Yamamoto S, Sobue T, Tsugane S. No association between fruit or vegetable consumption and the risk of colorectal cancer in Japan. Br J Cancer 2005;92(9):1782-4. doi: 10.1038/sj.bjc.6602566.

63. Vogtmann E, Xiang YB, Li HL, Levitan EB, Yang G, Waterbor JW, Gao J, Cai H, Xie L, Wu QJ, et al. Fruit and vegetable intake and the risk of colorectal cancer: results from the Shanghai Men's Health Study. Cancer causes & control : CCC 2013;24(11):1935-45. doi: 10.1007/s10552-013-0268-z.

64. Voorrips LE, Goldbohm RA, van Poppel G, Sturmans F, Hermus RJ, van den Brandt PA. Vegetable and fruit consumption and risks of colon and rectal cancer in a prospective cohort study: The Netherlands Cohort Study on Diet and Cancer. Am J Epidemiol 2000;152(11):1081-92.

65. Vulcan A, Brandstedt J, Manjer J, Jirstrom K, Ohlsson B, Ericson U. Fibre intake and incident colorectal cancer depending on fibre source, sex, tumour location and Tumour, Node, Metastasis stage. Br J Nutr 2015;114(6):959-69. doi: 10.1017/s0007114515002743.

66. Borch D, Juul-Hindsgaul N, Veller M, Astrup A, Jaskolowski J, Raben A. Potatoes and risk of obesity, type 2 diabetes, and cardiovascular disease in apparently healthy adults: a systematic review of clinical intervention and observational studies. Am J Clin Nutr 2016;104(2):489-98. doi: 10.3945/ajcn.116.132332.

67. McGill CR, Kurilich AC, Davignon J. The role of potatoes and potato components in cardiometabolic health: a review. Ann Med 2013;45(7):467-73. doi: 10.3109/07853890.2013.813633.
